# Adding Value to Cassava Genetic Resources Conserved at CIAT—Part I: A Review of Fifty Years of Collection, Conservation, Characterization and Distribution

**DOI:** 10.3390/plants15131981

**Published:** 2026-06-26

**Authors:** Clair H. Hershey, Ericson Aranzales R., Gustavo Jaramillo O., Norma C. Manrique-Carpintero, Monica L. Velez-Tobon, Peter Wenzl

**Affiliations:** 1 Independent Researcher, Formerly, Cassava Program, Centro Internacional de Agricultura Tropical (CIAT), Recta Cali-Palmira Apartado Aéreo 6713, Cali 763537, Colombia; 2 Genetic Resources Program, Centro Internacional de Agricultura Tropical (CIAT), Recta Cali-Palmira Apartado Aéreo 6713, Cali 763537, Colombia

**Keywords:** ex situ conservation, in vitro slow-growth, cassava collection, genebank, cassava conservation, cassava landraces, genetic resources distribution, wild *Manihot*

## Abstract

Improved varieties strengthen cassava’s roles as both a food security staple and a versatile industrial raw material across the tropical regions where it is produced. In support of this effort the International Center for Tropical Agriculture (CIAT) curates the world’s largest cassava germplasm collection at its Cali, Colombia, research center. Since the first collection expeditions in 1969, a primary focus was to assemble and conserve the diversity from the crop’s center of origin in the American tropics. Later additions expanded representation from Asia and Africa as secondary centers of diversity. The collection now consists mainly of landraces (about 5000 accessions), bred lines from CIAT (375) and from partner institutions (253), and related wild *Manihot* (377 accessions from 23 species or subspecies). Landrace diversity originated and evolved almost entirely through occasional farmer selections from seed-derived plants, which were subsequently conserved clonally over many generations. Secure ex situ conservation, first as a field collection and then in a slow-growth in vitro system, gave priority to pathogen testing and reliable culture and exchange methods. Cryopreservation research is ongoing to achieve added security and efficiency. CIAT extensively characterizes accessions through morphological, biochemical and molecular criteria. As a core goal, the collection has been a foundation for genetic improvement of the crop globally. This paper provides perspectives on the future management and use of the collection in the context of the recently established Future Seeds genebank facilities in Colombia, and new tools and technologies that support more effective conservation, evaluation and use.

## 1. Introduction

CIAT’s activities in collection, conservation, characterization, evaluation and use of crop genetic resources, particularly for tropical forages, beans and cassava, date from the earliest years of the center’s founding in 1967. In 2017, CIAT published a detailed 50-year, center-wide history [1], including some details on the crop and forage species collections. Schultze-Kraft et al. [2] and Debouck et al. [3] published histories of the forage and bean collections, respectively. The current work covers a similar history for the *Manihot* species collections at CIAT.

While CIAT gives highest priority to the cultivated species *M. esculenta*, it also maintains a small collection of wild relatives, among the approximately 100 species of the genus *Manihot*. The roles of the germplasm collection have continually evolved from the early years—from initially being a direct source of recommended varieties, to parents in breeding populations where the focus is on more targeted and specialized traits to address arising problems and opportunities. These traits may include, for example, resistance to new pests and diseases, specialty quality traits, or physiological traits associated with productivity under new climate conditions and new production or processing systems. The 50 years of the cassava collection’s history are just the beginning of CIAT’s commitment to its conservation in perpetuity, for continued human and environmental benefit. The body of the paper is organized topically to cover collection, conservation, evaluation and use, across more than half a century of the life of the genebank. Table S1 provides a broad-sweep chronological overview of activities and outputs over this history [4,5,6,7,8,9,10].

Elements of this paper are extracted from a comprehensive internal CIAT historical review by C. Hershey and co-authors covering the 50-year history of the cassava germplasm collection and the use of landrace varieties in breeding.

## 2. Cassava as a Global Crop

Since its domestication up to 7–10,000 years ago in the neotropics, this resilient species—*Manihot esculenta*— has sustained livelihoods with its reliable production of starchy roots. After the arrival of Europeans in the Americas in the late 15th century, cassava’s importance expanded from its evolutionary homeland into both Africa and Asia. However, only in roughly the past 50 years has the crop evolved from being mainly a subsistence, poor farmers’ crop—often grown on lands marginal for soil fertility and/or acidity, and drought challenges—to a multi-purpose crop that supports a wide range of agricultural, consumer and industrial needs [11]. It now ranks third in food crop production in the tropics and seventh globally. It is especially notable as a critical source of food calories in Africa, where Nigeria, DR Congo and Ghana account for over half of the continent’s production. In the Americas, Brazil alone produces nearly 70% of the region’s total. Thailand, Indonesia and Vietnam dominate as producers of starch for industry, with very fast growth in Cambodia and Lao PDR [12].

**Figure 1:**
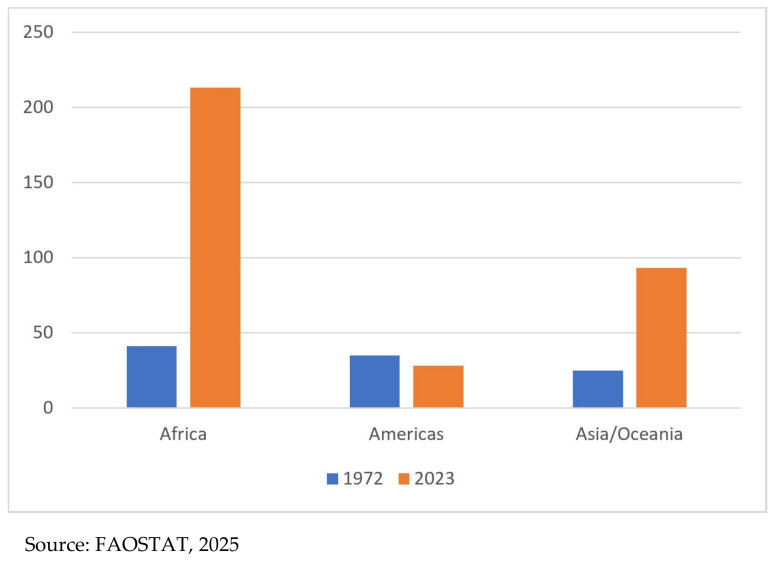
Change in global cassava production (metric tons fresh roots) 1972–2023, by region.

Production shifted dramatically in the last half century—most notably a remarkable five-fold increase in Africa from 1972 to 2023 (Figure 1). This was primarily due to the increased area planted, but also modest yield improvements. Just four countries (Nigeria, DR Congo, Thailand and Ghana) account for over half of current global production. Much of the research effort in Africa over the past five decades has focused on minimizing yield losses from the widespread cassava mosaic disease, and, more recently, the cassava brown streak disease, currently limited to eastern Africa. In Asia, production over the same period has more than tripled, resulting from both increased yields and area planted. Conversely, in the center of the crop’s origin, the Americas, production has fallen by 22%, with yield increases somewhat offsetting the reduced area planted [12].

Cassava stands out among crops that are well-positioned to adapt to climate change and to provide high yields under modest inputs. The fact that it is most important in areas of the world where people are least able to absorb instability in income or food supply places a special burden and responsibility to invest in more resilient, more productive, more consumer-oriented varieties. The opportunities to exploit these and other features will center around the integration of improved production practices and genetic resources. The latter includes collection, conservation, evaluation and the use of ex situ collections to develop varieties for changing needs.

## 3. The Foundations of Current Cassava Genetic Diversity

### 3.1. Impact of Natural Selection and Human Influences

Understanding how landrace varieties arise provides important keys to their characteristics and potential contributions to the development of new varieties through breeding. Evolutionary genetics, genome–environment interactions, and ethnobotanical evidence inform this understanding. Early cultivation reduced diversity compared to ancestral species around loci favored by humans [13]. After domestication, landraces diversified through a combination of local adaptation to climate, soils, biotic pressures and farmer-mediated selection for agronomic, culinary and cultural traits [14]. Nature and farmers together have applied a wide range of selection forces toward the slow and continual genetic changes in landrace varieties during cassava’s long history of domestication and traditional cultivation. There are several ways that we can get insights into these selection forces:

- Characterization of the physical and biological environments where cassava was historically grown. This provides some understanding of the adaptations and resistances that were required for the crop to succeed.
- Characterization of the end-uses and trait preferences, including through archeological findings, historical records, and current uses in traditional systems.
- Surveys, participatory research and other forms of interaction with farmers, processors and consumers to understand current-day and future varietal preferences.
- Measurement of cassava genetic diversity through field and laboratory evaluations across a range of environments and uses.
- Molecular characterization (especially direct measures of DNA polymorphisms) and studies of associated genotypic and phenotypic variation, as well as evidence for patterns of crop evolution and distribution.

#### 3.1.1. Climate and Agroecosystems

Although cassava appears to have been domesticated in a rainforest environment [15], the crop, like many of its wild relatives in the genus *Manihot*, has adapted especially well to seasonally dry areas, broadly defined by distinct rainy and dry seasons across the year. Brief periods of drought stress during the normal rainy season can create severe constraints for many crops, especially during sensitive developmental events such as pollination. However, cassava is well-known for its ability to tolerate and recover from brief or even extended drought periods, providing a primary driver for its popularity in seasonally dry areas across the tropics.

This species adaptability facilitated cassava’s spread from the humid Amazonian and Orinoco rainforest habitats—west and north to the inter-Andean valleys of Bolivia, Peru, Ecuador, and Colombia; to Meso-American and the Caribbean; and east to the semi-arid Caatinga region of northeast Brazil, where the crop can be found in areas with as low as 500 mm/yr rainfall [16].

Although the species as a whole retained its intolerance to low temperatures (especially sub-freezing), over millennia, some landraces gradually adapted to the mid-altitude tropics, and even up to a limit of around 2000 m above sea level (masl). Cassava also spread to the extremes of its latitudinal temperature adaptation, to the northern and southern hemisphere subtropics. Production became especially significant in the subtropics in southern Brazil, Paraguay and Argentina. This distribution was probably relatively recent compared to the timeframe for domestication in the warm tropics [16,17]. The specific physiological adaptations for response to daylength or to low winter temperatures have not been well-studied. In general, adaptation to low winter temperatures seems to have been more a function of crop management rather than genetic adaptation, e.g., harvesting before frost occurs and storing stems until the following warm season [18].

#### 3.1.2. Biotic Environments

As a vegetatively propagated crop, cassava is especially vulnerable to many pests and pathogens that can be transmitted from one crop cycle to the next via stem planting pieces. Further, because of a long growing season of up to a year or longer, there is an extended opportunity for attack by pests or pathogens with varying seasonal adaptations. Nonetheless, historically and mainly prior to the intensive research on cassava begun by the International Agricultural Research Centers, cassava was often referred to as a rustic crop. FAO [19] summarized this sentiment in their 1964 Yearbook: “Cassava grows reliably under drought and poor soils and is little affected by the pests that damage other staples.”

It was thought to be highly tolerant to biotic-induced stresses in the environment, and to require little care to reliably produce reasonable yields. This perception changed dramatically as data accumulated from systematic research. One of the revelations was the response when cassava landraces were moved from their native area of production in traditional systems to completely different environments. This mass movement across diverse environments was first carried out by CIAT soon after establishment of the cassava collection in Colombia. The process of introducing traditional cassava landrace varieties into new environments provided some of the first broad evidence that cassava could be highly vulnerable to damage from many pests and diseases.

The Americas—the crop’s evolutionary homeland—are also the center of diversity of pests and diseases. The literature reports over 200 species of arthropods attacking cassava [20]. Among them, various species of mites and whiteflies are the most devastating. Hillocks and Wydra [21] list some 40 bacterial, fungal and nematode pathogens. Nearly any cassava plantation anywhere in the world will probably demonstrate some level of infestation or infection with pathogens, but most are not serious. However, some diseases such as cassava bacterial blight, cassava mosaic disease, witches’ broom disease, or various root rot pathogens, among others, can be devastating if inoculum load and weather conditions are suitable, if vectors are present (for some pathogens), and the host plants lack resistance. The viruses tend to be more continent-specific but there is continuing risk of introductions to new areas [22]. Most notably, the cassava mosaic virus, a Begomovirus, is widespread in Asia and Africa but so far has not been reported in the Americas.

Within the Americas, it can be assumed that many of these pests and pathogens have been associated with cassava for centuries, or even millennia, and have certainly impacted the evolution of resistance mechanisms in landraces selected by growers.

#### 3.1.3. Production Systems and Soils

Key crop management practices important to the evolution of cassava’s genetic diversity are likely to include:

- Scaled harvesting over extended periods of time. With the normal rapid physiological deterioration of cassava roots after harvest, a common practice was to harvest small numbers of plants as needed for household use. In this sense, cassava does not have a well-defined maturity period. This practice would have favored the accumulation of alleles that contributed to achieving and maintaining good pre-harvest root quality for extended periods of time.
- Intercropping of multiple species along with cassava was probably the norm in most traditional areas of production. This practice would have driven selection for traits such as canopy suitability (e.g., competition for light and weed control), and system adaptation to competition for water and nutrients.
- Low use of added nutrients. In traditional systems, soil nutrients would be optimized mainly with crop rotations, use of fallow periods, and intercropping of compatible species. In general, under these conditions, cassava would have been selected for its capacity to extract/use nutrients efficiently. Cassava is better adapted than many crops to the low-fertility and highly aluminum-saturated, weathered soils of the Amazon rainforest and tropical savannas, where many crops will not survive without significant pH amelioration.

#### 3.1.4. Cassava Processing and Use

Each stage of the cassava food chain evolved in response to specific grower and consumer preferences, which contributed substantially to landrace diversity. Starch content and starch quality are key determinants of varietal suitability for particular uses, but acceptable ranges differ by product type, consumer group, and region. Consequently, landrace varieties exhibit broad variation in starch levels and functional properties. Environmental effects are also pronounced: extended drought or high temperatures typically reduce root starch content, and levels may decline sharply when reserves are mobilized for foliage regrowth, such as after rains following drought or after pest- or disease-induced defoliation.

Preferences for fresh consumption versus processed products strongly shaped selection. Root cyanogenesis is one of the most important post-harvest differentiating traits. All cassava varieties produce cyanogenic glucosides—primarily linamarin—in roots and leaves. Varieties with low cyanogenic potential (CNP), generally defined as ~50 ppm CN^−^ or less for fresh roots, are considered safe after simple boiling [23]. Higher CNP varieties require more complex processing (e.g., grating, fermenting, mashing, roasting) to reduce cyanogens to safe levels. The rationale for farmer preference for high-CNP types in some systems is still debated. Pest and disease resistance has been proposed but lacks strong empirical support. Deterrence of wild foraging mammals—or even theft—appears more plausible. Farmers also commonly speculate, as noted in author experience and in the literature [24], that high-CNP types yield superior starch or flour (e.g., farinha, garri), although this has not been experimentally verified. Landrace varieties nonetheless span a wide CNP range (www.genesys-pgr.org/c/cassava (accessed on 25 September 2025)).

Cassava storage roots deteriorate rapidly after harvest under ambient conditions [25]. Within days, physiological deterioration begins, producing black streaks in the parenchyma. As deterioration progresses, roots become unacceptable for human consumption and eventually unsuitable even for feed or industrial use. Despite extensive research, genetic variation for reduced post-harvest physiological deterioration appears present but relatively narrow among landraces.

Given the diversity of cassava processing methods and end products, substantial variation in quality-related traits is expected in germplasm collections. The rapid expansion of starch, feed, energy, and other industrial uses is relatively recent. These markets have influenced breeding priorities but have not altered the genetic diversity of traditional landrace varieties that dominate collections.

Religious and cultural uses of cassava have been documented [26,27]. While noteworthy, these uses likely contributed only modestly to overall species-level diversity.

### 3.2. Effects of Reproduction Systems

Cassava’s dual reproductive system—sexual and clonal—has profoundly shaped the crop’s diversity. In general (with some exceptions), a cassava variety is a clone, and all plants within a variety are genetically identical. For monoclonal varieties, any individual plant can fully represent the variety for ex situ conservation. This contrasts with outcrossing crops such as maize, where each plant in an open pollinated population is genetically distinct and multiple plants must be sampled to capture allelic diversity.

At the same time, all known cassava varieties are highly heterozygous. Thus, every seed produced through sexual recombination—whether by self-pollination or crossing—is genetically unique due to random segregation of parental alleles. (Throughout the text, the term *seed* indicates botanical seed resulting from a pollination event, while the stem pieces used for clonal propagation are labeled as *stakes* or *stem pieces*, in contrast to the common use of the term *seed* in the literature or by growers to also refer to stem pieces). Seed production is the primary mechanism through which farmers generate new varieties within their existing varietal mixtures. Farmers may not understand the underlying process of genetic recombination but often recognize seedlings as potential new varieties and may deliberately propagate and protect them [28].

Seeds produced in farmers’ fields fall to the ground and may be consumed by animals, removed by insects or birds, deteriorate microbially, or germinate after a delay, for example, following burning in slash-and-burn systems [29]. Seedlings typically show lower vigor than plants from stem cuttings, which benefit from large carbohydrate reserves. Seedlings also tend to yield less due to competition and often produce a taproot—generally undesirable—rather than the multiple thickened roots that develop around a planted stem cutting.

Recognizing the potential of seedling-derived plants requires careful observation, as their full agronomic potential is expressed only after subsequent clonal propagation. Nevertheless, farmers have undoubtedly incorporated stem cuttings from seedlings into their planting material, both knowingly and unknowingly.

Modern breeding experience indicates that the probability of superior clones arising from random true seed production in farmers’ fields is low, especially where a single clone is cultivated over large contiguous areas, increasing the likelihood of effective self-pollination. The farmer “breeding” process is more effective when multiple varieties are planted in close proximity, enabling cross-pollination among unrelated genotypes.

A detailed study by Duputié et al. [28] showed that Amerindian farmers in the Guianas intentionally expand their varietal options through seed. Farmers often incorporate seedling-derived plants into the varietal group they most resemble. Skilled farmers recognize that first-generation seedling plants may have lower agronomic performance. The study also noted that landrace identity may erode over time as genetically distinct but morphologically similar seedling-derived clones are incorporated. These dynamics are important for designing collection and ex situ conservation strategies.

When most of CIAT’s cassava germplasm was collected in the 1960s and 1970s, this farmer-mediated seedling selection process was scarcely documented. More recent work [28,29] has substantially improved our understanding of how farmers consciously and unconsciously use seedlings to maintain diversity, and what “variety” means in farmer practice—and thus in genebank management. At the same time, the importance of this process should not be overstated. Across much of the Americas, seedlings likely played a gradual and relatively minor role in generating diversity for most farmers, even though, over longer evolutionary timescales, sexual recombination was the primary source of new varieties.

### 3.3. Distribution of Landraces from Centers of Origin

In general, throughout cassava’s early history, individual varieties arose and were maintained in relatively limited geographic areas—shared among farmers within villages and among neighboring villages. Less commonly, exchange occurred across broader regions, such as during migration and the development of new settlements. These dynamics meant that many thousands of distinct varieties were developed throughout the cassava production zones of the Americas. Many of them had relatively narrow adaptation to the specific conditions and end-uses in the area where they were first selected. Because of vegetative propagation, any given variety, during the process of normal clonal propagation, could not further genetically adapt to a new environment or a new use, such as happens readily with seed-propagated and cross-pollinated crops like maize. Ongoing studies based on genotyping of CIAT’s collection (discussed further in a later section) show a limited number of currently existing accessions that were widely spread geographically within the Americas prior to formal research activities, and some probably even to Asia [10,30].

Kistler et al. [31] shed light on cassava variety development and distribution through a wide-ranging study designed to understand how the specific landscape of cassava interfaces with clonal reproduction and selection. One of the major findings—affecting how we think about ancestral and current genetic diversity of landraces—was that all cultivated cassava worldwide, ancient and modern, shares large, identical haplotype blocks. They found that 82% of all landraces from the Americas have a second-degree or closer relative collected elsewhere. Varieties near the center of cassava’s origins (SW Amazon basin) tend to have spread more widely, with small genetic variations over time due to new varieties arising from seeds. Distant varieties, such as those in Meso-America or the Caribbean, were more genetically stable and spread less widely. The research also found that 59% of tested cassava genomes included non-*M. esculenta* ancestry, certainly a significant contributor to the trait diversity available for farmer selection.

### 3.4. Impact of Landrace Replacement by Bred Varieties for On-Farm Diversity

Farmers appear to have cultivated, processed and consumed cassava in much the same ways over many centuries and, in some systems, up to current times. For these traditional purposes and systems, demand for substantially different new varieties was limited. The introduction and application of science-based crop breeding technologies came into play only toward the middle of the 20th century—a relatively short time span to affect overall genetic diversity across most cassava-growing areas. Until the 1970s, nearly all farmers grew traditional landraces [11]. However, in that era, global efforts were underway, especially with the “Green Revolution” crops like wheat and rice, to breed high-yielding varieties that would replace traditional ones. It was an opportune time to prepare for the eventuality of new cassava hybrids significantly replacing traditional landraces. This foresight drove the establishment of a comprehensive collection of landraces from cassava’s center of origin, along with the knowledge about the crop that had been passed down over centuries by the caretakers of that diversity.

## 4. Collection and Acquisition

### 4.1. Planning for First Collections

CIAT’s 1969 Annual Report [32] articulated an ambitious objective for its newly established Roots and Tubers Program: “to explore and collect cultivars and related wild species of *Manihot* in the countries where variability is present, with emphasis in the primary centers of origin (Northern South America and Middle America), in order to establish a germplasm bank representative of the world’s variability.” The goal was to assemble a germplasm bank that captured the breadth of global cassava diversity. This mandate reflected both the scientific priorities of the time and the recognition that cassava, despite its importance as a staple crop, had received relatively modest research attention.

Prior to CIAT’s involvement, several countries maintained local or regional cassava collections—most notably Brazil, the Malagasy Republic, Uganda, Congo, Colombia, and India [8]. These collections were generally limited in scope, unevenly characterized, and often found outside cassava’s center of origin. As a result, they likely represented only a narrow fraction of the crop’s genetic diversity. CIAT’s strategy therefore centered on assembling a more comprehensive and evolutionarily representative collection, with particular emphasis on landrace varieties maintained by farmers.

To lead this effort, CIAT enlisted the expertise of Victor Manuel Patiño, director of the Botanical Garden of Valle and one of the region’s foremost botanists. Beginning in May 1969, Patiño initiated a two-year effort that would become foundational to global cassava genetic resource conservation. His early planning targeted cassava-growing regions throughout the Americas and the Caribbean, but logistical and phytosanitary constraints shaped the initial scope. In particular, Colombia’s concern over coffee rust prevented the introduction of Brazilian landraces for nearly two decades, until in vitro exchange methods and revised quarantine regulations enabled safer movement of plant material.

The first phase of collection in 1969 focused on Colombia, both to refine methodologies and because of ease of access. Patiño’s team collected 611 accessions from 20 departments, which were processed through Colombian quarantine and established at CIAT’s Palmira farm. In 1970, the effort expanded regionally, and by the end of that year the genebank held the following totals from the combined 1969–1970 expeditions [33]:

- Colombia—1884;
- Ecuador—123;
- Puerto Rico—60;
- Panamá—118;
- Peru—eight;
- Venezuela—330 (in quarantine in Bogota);
- Mexico—70 (awaiting shipment to Colombia).

### 4.2. Collection Procedures

#### 4.2.1. Type of Plant Material Collected

Throughout the first decade of CIAT’s cassava collecting, procedures were broadly consistent across countries and missions. Because cassava is vegetatively propagated, nearly all landrace accessions were collected as clonal stem cuttings rather than botanical seed. There is no evidence that collectors routinely sampled true seeds from farmers’ fields, and in vitro collection remained experimental until later decades. As a result, most landrace accessions in the genebank represent direct clonal equivalents of the varieties grown by farmers at the time of collection.

A small number of seed-derived introductions from Brazil—primarily from institutional collections—entered the genebank in the early years before in vitro exchange was available. These accessions have no direct clonal equivalents elsewhere and thus represent unique genetic material.

**Figure 2:**
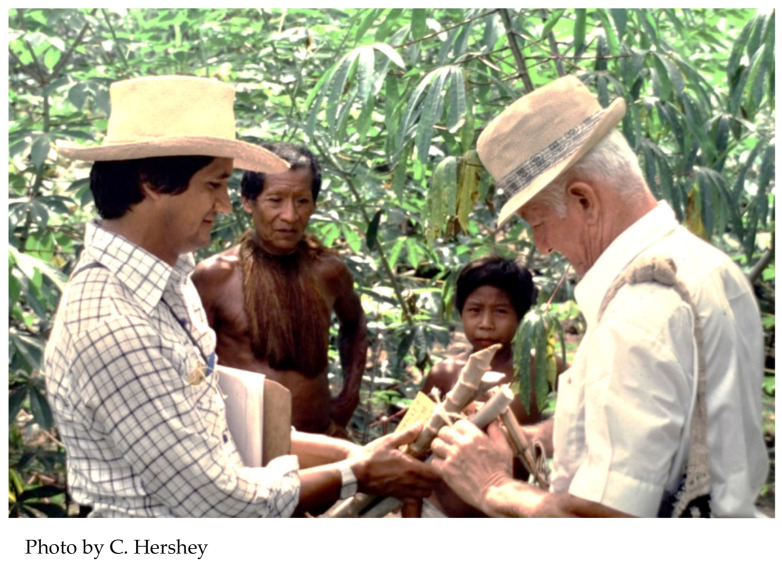
Alvaro Amaya (left) and Pablo Daza (right) from CIAT receive stem pieces from a Tikuna farmer (also Magüta: “people of the painted fish”) in Amazonas Dept., Colombia, during a collection expedition in 1979. The indigenous farmers of the Amazon basin developed some of the most important cassava diversity and willingly shared it for humanity through the CIAT cassava collection. Mr. Daza accompanied many of the earliest collection expeditions in 1969 and 1970.

Collecting stem cuttings requires careful logistical planning. Typical sampling involved cutting five to six stem pieces, each 50–60 cm long, usually from a few different plants of a farmer-identified variety (Figure 2). Bundles weighed a few kilograms, and collectors often traveled long distances in remote areas under challenging conditions. Despite these constraints, reports suggest that teams generally managed the material effectively, ensuring viability during transport and quarantine.

#### 4.2.2. Passport Information and Accession Coding

From the outset, collectors recognized the importance of documentation, but the specific types of information most valuable to future users—particularly breeders—were not yet fully understood. As a result, passport data for most early accessions are rudimentary. Typical records include approximate collection location (often described as distance from a village), the farmer’s name (usually the male head of household), and the local variety name. Genebank curators retrospectively added geographic coordinates based on collectors’ descriptions, making it impossible today to identify most specific farms of origin (www.genesys-pgr.org/c/cassava).

**Figure 3:**
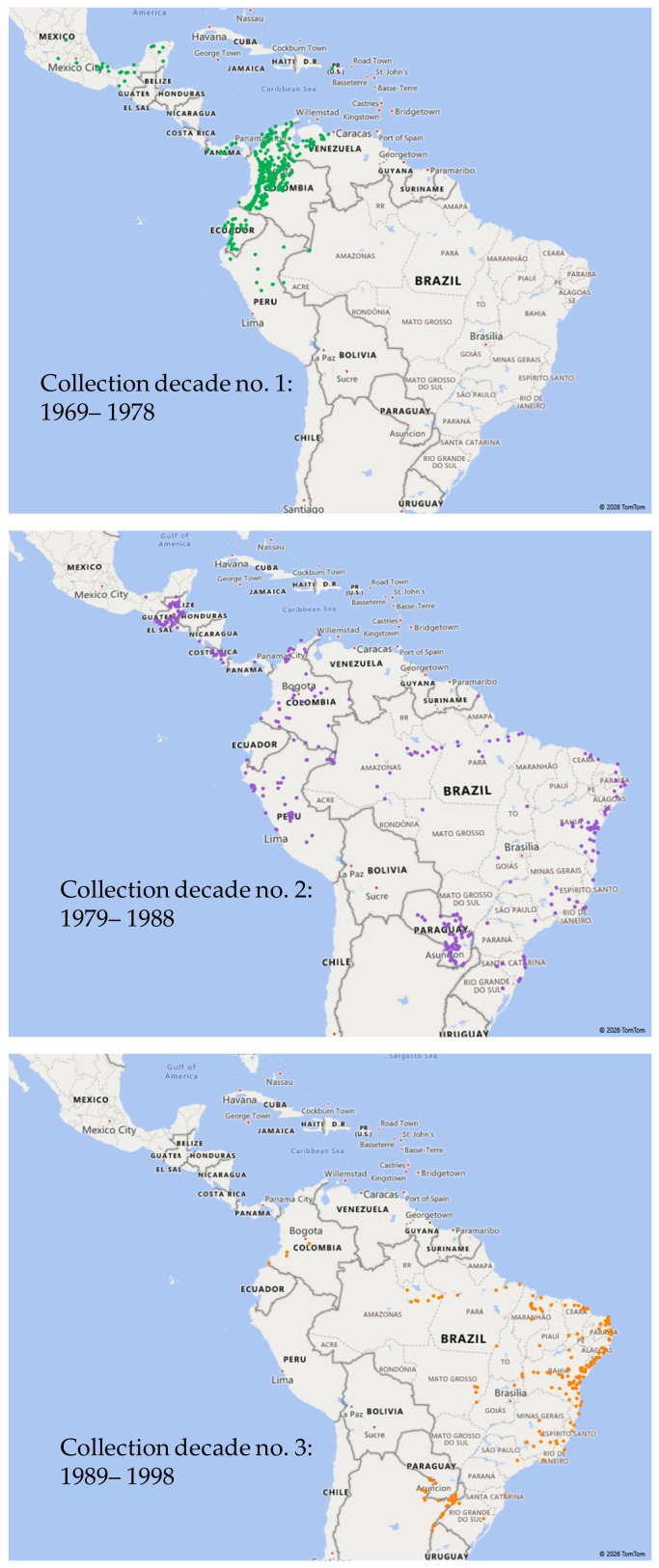
Collection sites for landrace varieties added to the CIAT cassava collection over three decades, beginning with initial genebank establishment in 1969. Some accessions acquired from institutional genebanks do not have geo-referenced collection sites and therefore are not included here. Each dot represents a collection site. Source: www.genesys-pgr.org/c/cassava (accessed on 10 March 2025).

Collectors recorded site altitude for roughly half of the landrace accessions. Although cassava’s adaptation to lowland versus highland environments is not precisely defined, experience suggests that materials collected below and above roughly 1200–1400 masl often differ in temperature adaptation. Only 11 accessions were collected above 2000 masl, all from Colombia (Table 1). Figure 3 maps known collection sites from the first three decades, with details available in CIAT’s databases (www.genesys-pgr.org/c/cassava (accessed on 12 March 2025)).

Upon entry into Colombian quarantine, each accession received a standardized CIAT code: the prefix M (for *Manihot*), followed by a two- or three-letter country code and a sequential number (e.g., MCOL 1, MECU 1, MVEN 1). This system remains foundational to CIAT’s germplasm nomenclature. (In the literature and in databases, the two- or three-letter country codes may be in upper or lower case. Current standard use is for all upper case. More recently (the date is not well-documented) the letter M was dropped, since it does not add any information about the accession).

In 1982, an IBPGR working group proposed an expanded passport format [34], emphasizing farmer knowledge, production conditions, and collectors’ observations (Figure S1). Under this new scheme, collectors would ask farmers to describe an array of traits—yield, earliness, extended harvest capacity, pest and disease resistance, and root quality for specific uses [34,35]. These more detailed formats were applied in two major Colombian collections: Amazonas (COL 2068–2214) in 1979 and Llanos Orientales (COL 2266–2419; COL 2520–2624) in 1986–87 [36].

Despite the value of expanded passport data, the cost and complexity of recollecting previously sampled areas would be high relative to expected benefits. Moreover, early collectors often overlooked the knowledge of women farmers, who in many traditional systems are the primary custodians of varietal diversity. Male collectors typically interacted with male farmers, limiting the capture of women’s expertise. Collections led by Jan Salick in Peru [27] were notable exceptions.

Collecting missions are labor intensive, and time constraints often limited opportunities for in-depth interviews. As a result, much farmer knowledge was not recorded. Nevertheless, the secure conservation of these accessions now enables targeted evaluation to recover insights that were not captured during collection.

### 4.3. Continuing Collection and Acquisition Beyond the Early Genebank Establishment

**Figure 4:**
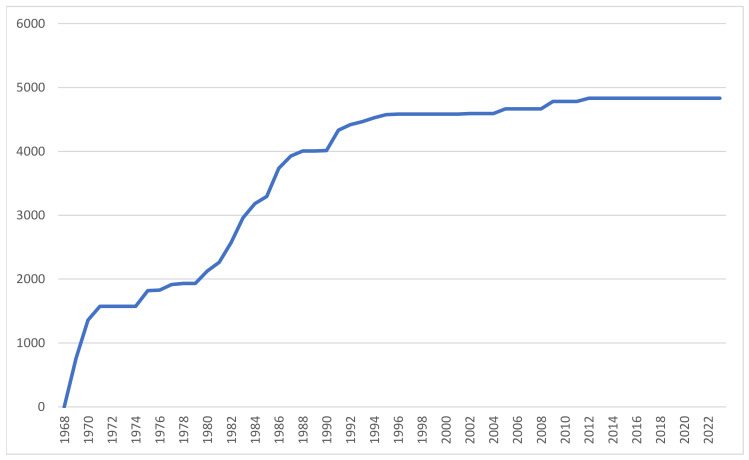
Cumulative growth across years of CIAT cassava collection, with number of successful introductions of landrace varieties, where “successful” refers to accessions that have survived and have been retained to the present time.

The Patiño expeditions of 1969–1970 remain the single largest contribution to CIAT’s cassava genebank. Accessions from these two years form roughly one third of the current landrace collection—meaning that the first two years yielded about half as many landraces as the next five decades combined (Figure 4). After this initial surge, new accessions came from three primary sources:

- Small to medium-sized expeditions designed to fill geographic or ecological gaps.
- Opportunistic collections by scientists traveling for other purposes who encountered varieties not represented in the genebank.
- Acquisitions from national or regional programs, including landraces and hybrids. Although Genesys distinguishes these categories, incomplete historical records sometimes complicate classification.

Early annual reports [32,33] show that CIAT envisioned a gradual, systematic expansion of the collection after the initial push. The long-term goal was an ex situ repository representing a high proportion of global cassava diversity. However, early collectors lacked tools to quantify diversity or identify gaps, so priorities focused on sampling major production areas rather than on detailed genetic assessments.

A secondary objective emerged, to explore diversity in marginal or atypical environments where unique selection pressures might have shaped cassava evolution. These included:

- Seasonally dry or semi-arid regions;
- High-altitude zones (up to or slightly above 2000 masl);
- Higher latitudes—where photoperiod and low winter temperatures differ markedly from the tropics;
- Moderately higher pH soils;
- Areas with significant pest or disease pressure.

Globally, Hershey [8] estimated that at least 5000 more landraces would be needed in order to fully capture cassava’s genetic diversity. Priority regions included Bolivia, Brazil, Colombia, the Guianas, Haiti, Nicaragua, Peru, and Venezuela. In retrospect, insufficient attention to the Guianas—historically, a crossroads of cultural exchange among the Caribbean, Amazon, and Orinoco regions—may be one of the more significant gaps in early strategies.

Beyond field expeditions, CIAT often collaborated with national and international partners to introduce accessions from existing collections. After 1971, this became the primary mode of acquisition. Table 2 summarizes additions by decade and by germplasm type, with Table S2 detailing regional and institutional origins.

### 4.4. Asia and Africa as Secondary Sources of Diversity

Although cassava’s evolutionary origins lie in the Americas, important secondary centers of diversity arose in Asia and Africa. From an evolutionary standpoint, it is unlikely that a substantial number of new mutations of value accumulated in these regions within the few centuries since cassava’s introduction to Africa and later spread to Asia. Nevertheless, farmer selection, environmental pressures, and local preferences have shaped allele frequencies, producing regionally adapted diversity. As global genotyping capacity expands, one of the most illuminating developments will be the ability to identify clonal equivalents across continents, reconstructing historical dispersal pathways and clarifying how American landraces were transformed through farmer selection in Africa and Asia [10].

Historically, quarantine procedures shaped the flow of germplasm from these regions to CIAT. Introductions from most of Asia (except for India) were relatively straightforward until the discovery of the Sri Lanka strain of cassava mosaic disease (CMD) in 2015 [37], which later spread across several countries and complicated international movement of cassava material. Introductions from Africa were more challenging from the outset, as CMD was already widespread on the continent when CIAT and IITA were founded. As a result, early introductions from Africa to CIAT were primarily hybrids developed at IITA for CMD resistance rather than representative samples of African landrace diversity.

To ensure phytosanitary safety, CIAT and IITA collaborated with the Scottish Crop Research Institute (SCRI) in Dundee, which served as an intermediate quarantine facility. The SCRI conducted additional virus indexing—especially for CMD—before forwarding material to Colombian authorities. CIAT then performed its own pathogen testing prior to field establishment [38,39]. These procedures enabled the safe introduction of valuable resistance sources while minimizing the risk of introducing pathogens into the Americas.

### 4.5. Cassava Landrace Density as an Indicator of Local and Regional Diversity

The literature reports a widely varying number of varieties that individual farmers typically grow (on-farm landrace density)—ranging from only a few varieties to several dozen [26,27,40]. Landrace density can also be assessed at the regional scale, providing insight into how diversity is structured across landscapes. Using CIAT’s collection records (www.genesys-pgr.org/c/cassava (accessed on 16 February 2025)), we estimated varietal density at several registered collection sites, based on the following assumptions:

- Each site represented a single farm or a small cluster of a closely associated set of farms.
- Collectors sampled all varieties recognized as distinct by farmers or by collectors themselves.
- Varieties obtained from institutions (e.g., experiment stations or schools) did not influence the likelihood of collecting the same varieties from farmers in the region.

Brazil could not be included in this analysis because most Brazilian accessions lack geolocation data. Similar limitations apply to smaller collections from Cuba and Costa Rica. Despite these constraints, the dataset provides meaningful insights.

Table 3 summarizes varietal density across nine countries, covering 2318 accessions from 1017 sites—approximately half of the landraces in the CIAT collection. The country-level averages suggest that most farmers typically grow only one to three varieties. Across the Americas, the mean is 2.3 varieties per site. Only Colombia and Paraguay include sites where more than ten varieties were collected.

Colombia’s detailed collection records allow deeper analysis. In several departments dominated by indigenous communities, varietal density is notably higher (Table 4). The most striking example is a site in Guainía, a remote region in the Amazonian basin, where collectors recorded 36 putative varieties—the highest number from any single site. Guainía also had the highest department-level average, with 15.8 varieties per site across nine sites.

These patterns align with expectations: regions closer to the Amazon basin, where shifting cultivation and traditional farming systems are still prevalent, tend to keep higher varietal diversity. In such systems, diversity is valued for agronomic, cultural, and culinary reasons. In contrast, more commercialized systems typically rely on fewer varieties.

Overall, these findings suggest that breeding programs do not necessarily need to supply farmers with many varietal options to achieve impact. In most regions, farmers are accustomed to growing only a few varieties, although exceptions exist in highly traditional systems.

### 4.6. Collector Perspectives and Practices Enrich the Genebank

Most CIAT collectors recorded only essential passport information, but a few provided unusually rich contextual detail. Among them, Dr. Mario Mejía Gutiérrez stands out. He led five major collection expeditions in Colombia in the late 1980s, supported by the International Board for Plant Genetic Resources (IBPGR) and CIAT [36]. These missions targeted areas not covered during the Patiño expeditions of 1969–1970, particularly regions of high indigenous presence and ecological diversity.

Dr. Mejía documented socioeconomic contexts, cultural practices, and historical patterns of migration and trade. His reports describe how river networks facilitated the movement of cassava varieties, shaping diversity across regions. This level of detail offers valuable insights into the processes that generate and maintain diversity and offers a model for how collection missions can integrate ethnobotanical, ecological, and historical perspectives.

### 4.7. Molecular Tools as a Support for Planning and Analyzing Collections

Traditional assumptions about farmers’ ability to distinguish cassava varieties have been challenged by molecular evidence. While some communities keep highly accurate vernacular classification systems, others show substantial discrepancies between farmer-identified names and underlying genotypes. To address this, CIAT’s Cassava Genetics section developed an economical SNP fingerprinting protocol using a 96-marker panel (SNPY CHIP) capable of differentiating thousands of genotypes with high confidence.

The first large-scale application of this tool characterized 173 landraces from indigenous communities in Colombia’s Amazonas Department, in collaboration with the Amazonian Institute of Scientific Research (SINCHI) [41]. The results revealed substantial synonymy and homonymy, underscoring the importance of molecular verification.

A later study in the Cauca Department surveyed 37 households and found that nearly 75% of farmers believed they were growing improved varieties [42]. Molecular analysis showed that only 10 households actually were. Conversely, 10 households who believed they were growing only landraces were in fact cultivating improved materials. These findings highlight the difficulty of relying solely on farmer recall or vernacular names for varietal identification.

In Vietnam, Ocampo et al. [43] combined farmer interviews with SNPY CHIP analysis across 1570 samples from six agroecological zones. Farmers reported 97 distinct variety names, while molecular analysis showed 85 unique genotypes—a relatively modest 12.4% overestimation. This contrasts with many regions where discrepancies are far larger.

Together, these studies demonstrate the essential role of molecular tools in:

- Identifying unique genotypes;
- Guiding collection strategies;
- Validating adoption studies;
- Assessing genetic diversity within and across regions.

As genotyping becomes more accessible, it will increasingly support both targeted collecting and more accurate curation of existing germplasm.

### 4.8. Wild Manihot Species Acquisition

Wild *Manihot* species face substantial risks of genetic erosion, primarily due to expanding human activity—agriculture, forestry, and urbanization—into native habitats. Climate change is expected to intensify these pressures, particularly because many wild species occupy highly specific ecological niches and are sparsely distributed across challenging environments. The greatest concentration of wild *Manihot* diversity occurs in central Brazil’s Cerrado region, an area that has undergone rapid agricultural expansion since the 1970s. A striking example of habitat loss is documented in Brazil: of 41 wild *Manihot* habitats surveyed in the late 1970s, only one remained intact 25 years later [44].

CIAT’s early vision, as reflected in the 1969 Annual Report [32], included the conservation of wild relatives as a safeguard against genetic erosion and as a potential resource for breeding. However, systematic collection was constrained by limited taxonomic clarity. A modern monograph on *Manihot* was not published until 1973 [45], meaning that early planning relied on scattered and outdated literature.

With roughly 100 wild species distributed across remote and often inaccessible areas, and with limited funding available, comprehensive collecting has rarely been feasible. Preliminary work by CIAT’s Biotechnology Research Unit (BRU) and Genetic Resources Program (GRP) began in the early 1980s, but significant introductions of wild species into the genebank did not occur until the 1990s. Renewed interest was driven in part by studies highlighting the potential value of wild species for breeding [46] and by Antonio Allem’s influential hypothesis that cassava originated in the southwest Amazon basin from *M. esculenta* subsp. *flabellifolia* and subsp. *peruviana* [15].

Between 1992 and 1995, CIAT collaborated with Allem and the International Board for Plant Genetic Resources (IBPGR) to introduce wild species accessions, primarily as true seed (Table 5). Some seeds were difficult to germinate and required embryo excision and sterile culture. Because regeneration cycles sometimes involved seed propagation, certain accessions may now differ in allelic composition from the original collections.

As with cultivated cassava, CIAT no longer maintains a field collection of wild species. Nonetheless, the accessions introduced during the 1990s are an important, if incomplete, safeguard against the loss of wild *Manihot* diversity.

### 4.9. Breeding Lines and Genetic Stocks as Part of the Genebank

Elite breeding lines are valuable genetic resources, but they are vulnerable to loss unless explicitly conserved. CGIAR genebanks operate under a shared policy framework that allows the incorporation of experimental breeding lines, although criteria and procedures vary by center. For cassava, in vitro conservation is often the most effective method, and CIAT’s Genetic Resources Program (GRP) provides the necessary facilities and expertise.

Beginning in the mid-1980s, CIAT breeders and the GRP agreed to incorporate elite breeding lines into the genebank [47]. To qualify, a line typically had to:

- Complete testing through the full breeding pipeline, from seedling evaluation to replicated multi-environment trials over at least two years;
- Demonstrate superiority in key traits such as plant architecture, yield, dry matter content, and pest or disease tolerance.

These in vitro stocks serve multiple purposes:

- A pathogen-free, genetically verified source for multiplication of planting material;
- A reference standard for confirming varietal identity in breeding nurseries or farmers’ fields;
- A clean source for distribution to other genebanks or breeding programs;
- A temporal benchmark for assessing genetic gains over time.

However, the number of elite breeding lines increases steadily, raising concerns that, over time, they could consume a disproportionate share of genebank resources. Consequently, CIAT discontinued the routine addition of new elite lines from its own breeding program in the mid-1990s. New introductions from partner breeding programs continue, especially of released varieties. Major contributors include programs in Brazil, Colombia, Indonesia, Nigeria, and Thailand.

Genetic stocks—such as mapping populations—may also be incorporated into the genebank, depending on objectives and operational principles. For example, in the mid-1990s, CIAT added a mapping population to ensure broad availability to participants in the Cassava Biotechnology Network (www.genesys-pgr.org/c/cassava (accessed on 6 February 2026)).

### 4.10. Current Status of the CIAT Cassava Collection

In 2008, the Global Crop Diversity Trust convened cassava genebank experts to develop a global conservation strategy for *Manihot* [8]. The resulting report estimated that CIAT conserves approximately 55% of global cassava diversity. With the increasing availability of genotyping data from CIAT, IITA, and other partners, these estimates will likely be refined.

As of mid-2025, the CIAT cassava collection (*M. esculenta*) included the components listed in Table 6, totaling 5574 accessions (losses are addressed in a later section). This collection—assembled through systematic expeditions, opportunistic sampling, international collaboration, and targeted acquisition of breeding lines and wild relatives—is considered to be the most comprehensive repository of cassava diversity worldwide.

## 5. Pest and Pathogen Management as a Fundamental Conservation Concern

Many cassava pathogens, including viruses, bacteria and fungi, are readily transmitted from one generation to the next via clonal propagation. Conversely, mites and insects are controlled relatively easily with the protocols of in vitro systems. For any genebank, optimum phytosanitary status of accessions is fundamental for good management. Not only do pests and pathogens present a threat to the goal of conservation in perpetuity, but they also critically impact the possibility of sharing accessions with interested parties, especially internationally—a core function of the CIAT genebank. From the relatively basic tools of 50 years ago to the molecular diagnostics of today, there is now a high assurance that any accessions currently conserved and offered for exchange are free of pathogens of quarantine significance.

### 5.1. Plant Health Considerations for Early Establishment of the Collection

All early cassava collections (through the 1970s) were in the form of mature stem cuttings. Aware of the importance and dangers represented by introduction of pests and pathogens, CIAT cooperated closely with the plant pathologists of the Instituto Colombiano Agropecuario [Colombian Agricultural Institute] (ICA) to develop phytosanitary protocols. ICA stipulated that introductions from countries outside of Colombia would be quarantined in an insect-free and specially adapted greenhouse at the ICA-Tibaitatá station near Bogotá, a region where no commercial cassava is cultivated due to the high altitude.

To inactivate potential viruses, cuttings were treated at 52 °C for 20 min, or at 50 °C for 60 min, with hot water. Although some viruses, mycoplasma-like organisms and bacteria could apparently be inactivated by thermotherapy, repeating the treatment was deemed necessary to attempt, as much as possible, a completely virus-free stock. After the treatment, the cuttings were protected with a fungicide to avoid root rots and stem rots during sprouting [32,33].

Despite these early measures, in 1970, the newly established collection in the CIAT station began showing signs of cassava bacterial blight (CBB). This was a potentially devastating development. CBB was known to be highly damaging in susceptible clones, and most accessions appeared to be susceptible. In addition to causing defoliation and lower yields, the bacteria became systemic and were passed from one clonal generation to the next via infected stem pieces. Difficulty in conservation and propagation, along with quarantine implications for the distribution of germplasm, could negate many of the potential benefits of the collection. Fortunately, CIAT pathologists developed effective protocols to eradicate CBB through careful selection of symptom-free planting material and an indexing system based on rooted sprouts. CIAT was able to keep the collection at headquarters CBB-free during the remaining span of over 30 years of the continuation of the field germplasm collection.

The CIAT station also has several other endemic pests and diseases, most of which cause low or moderate damage levels in locally adapted varieties. However, given the broad genetic diversity of the germplasm collection, pests and diseases showed highly variable effects across the range of introduced accessions. The Cassava Program (especially the Breeding, Pathology, and Entomology sections) frequently took advantage of ongoing low-to-moderate pest and disease pressure, and sometimes more severe outbreaks, to evaluate accessions for potential genetic resistance whenever possible. Chemical control, even in the field collection, was used expeditiously, since it could reduce or destroy biological control agents, thereby exacerbating infestation and damage by pests in the longer term.

One of the most important sanitary practices was to prohibit the entry of stem pieces into the headquarters station from outside sources, such as from breeding trials in other regions of Colombia, except in special cases with appropriate phytosanitary inspection and monitoring. The main pathogen targets were the causal agents of CBB, superelongation disease (SED) and the potential for various unknown pathogens, especially viruses.

### 5.2. Managing the On-Going Phytosanitary Status of the Accessions

From the time of genebank establishment up to current times, there has never been a breathing space, where the plant protection specialists were not busy with one or another of the phytosanitary threats to the dual functions of cassava germplasm conservation and exchange. Cuervo et al. [48] highlight the chronology of phytosanitary practices, based on the expanding knowledge base and tools associated with the diagnostics and cleaning of clonal materials.

The continuing challenges tend to be from viruses and a handful of diseases with unknown causal agents. A principal challenge for accession cleaning has been for cassava frogskin disease, first reported in 1971, and eventually becoming a major threat to cassava production in several areas of Colombia, Ecuador and Central America [49]. This disease would become a key reason for eliminating the field collection at the CIAT Palmira station in the early 2000s. By 1985, a highly sensitive graft indexing test was developed using the indicator variety *Secundina* (COL 2063)*,* implemented for the genebank and for sensitive experiments elsewhere. However, up to the current time, the causal agent(s) have not been confirmed. A possible complex of pathogens was implicated [49], but more recent research demonstrated that single torrado virus infections can cause the classical symptoms of the disease [50]. Fully resolving the causal agent(s) is critical to the collection’s future phytosanitary integrity.

The cassava witches’ broom disease (CWBD) is another devastating disease whose causal agent eluded scientists for many years. In 2013, a phytoplasma was reported to be associated with disease symptoms [51]. A more recent report identified a fungal pathogen as a more likely causal agent. Using a metagenomic approach, Leiva et al. [52] identified a member of the *Ceratobasidium* genus as strongly associated with CWBD symptoms. The fungus is present in diseased samples collected since the first recorded CWBD outbreaks in Southeast Asia and is transmissible by grafting. The fungus’ full role as causal agent is yet to be confirmed. CWBD and CFSD illustrate some of the continuing difficult challenges for accession management through better testing and cleaning protocols for key pathogens.

Toward this end, in 2009, CGIAR took the significant step of forming a system-wide working group on genebank plant health. The group is a network of germplasm health units that provide phytosanitary services to genebanks and breeding programs. Members work to improve the efficiency, quality, and safety of germplasm movement within and outside CGIAR, and to strengthen the capacity of national partners to comply with international phytosanitary standards. Genebanks holding major clonal collections—e.g., CIAT, IITA, Bioversity and CIP—form a subgroup that focuses on plant health issues and solutions for collections held as in vitro cultures. The working group functions were absorbed into what is now the CGIAR Genebank Accelerator (https://www.genebanks.org/about/).

## 6. Evolving Conservation Methods and Tools for Long-Term Security, Genetic Integrity and Analysis

**Figure 5:**
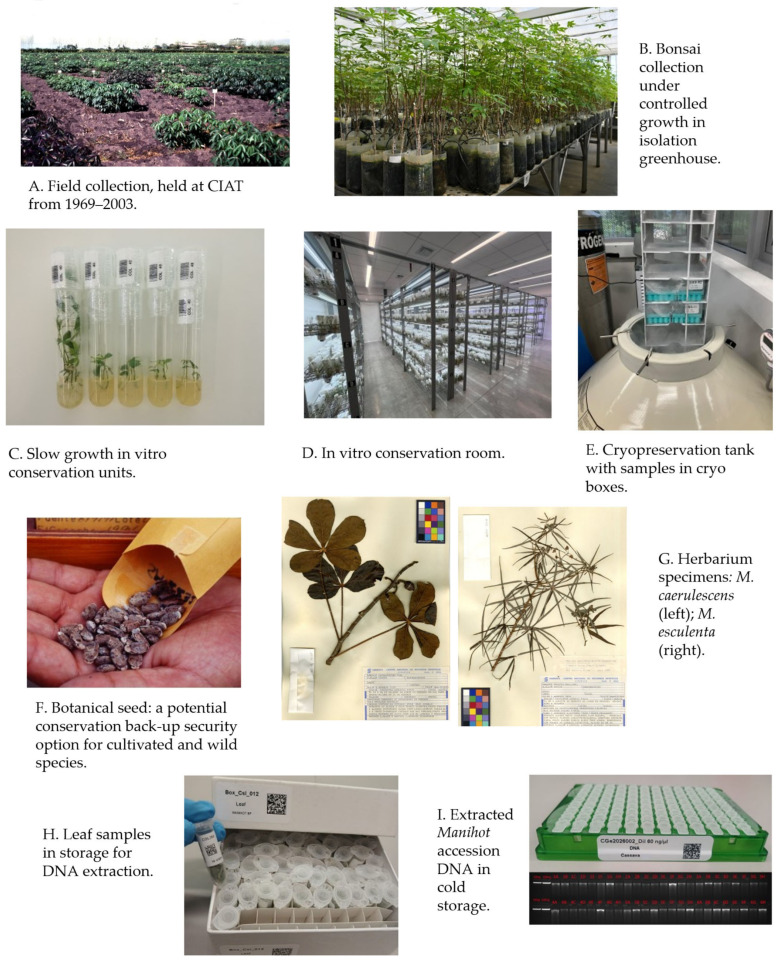
Illustration of multiple forms of conservation of *Manihot* germplasm and of biological specimens at the Future Seeds genetic resources facilities at CIAT. Photo credits: CIAT Communications (**A**,**B**,**F**); Juan José Gonzalez (**G**); Norma Manrique-Carpintero (**E**); Jessica Ospina (**H**,**I**); Monica Velez-Tobon (**C**,**D**).

Initially, when the collection was first established in the late 1960s, there was little thought given to options for clonal conservation other than as a field collection. By the mid-to-late 1970s, the promise of in vitro slow-growth conservation started to become a reality. At this point, there was the concept that an in vitro base collection should be the most secure system available for long-term conservation, and the field collection would be a working collection. By the late 1980s, cryopreservation evolved as a realistic option for cassava, and became the goal as a possible base collection, while both slow-growth in vitro and field accessions would form active or working collections (for characterization, evaluation, distribution and use in breeding). Although there has been great progress toward cryopreservation technologies, it is not yet fully operational at CIAT. Additionally, there are options for other forms of conservation that have emerged and are discussed here—including potted plants in the greenhouse with limited growth (bonsai method) and true seeds (Table S3) [53]. While not *germplasm* as formally defined, DNA samples and herbarium specimens complement the live accessions. Figure 5 illustrates the multiple conservation forms executed by CIAT.

### 6.1. The Field Collection

Space, labor, phytosanitary and multiple other challenges impact the viability and security of a field-based cassava collection. Guidelines for conservation and regeneration evolved into a system that balanced the overwhelming priority for safe conservation with the need to provide planting material for trials inside and outside the CIAT headquarters station.

Jaramillo [54] detailed the agronomic and phytosanitary practices that are fundamental to managing a field collection. Apart from the general management recommendations for cassava production (e.g., land preparation, soil fertility management, weed control), the field collection required some additional attention. In view of cassava being a perennial plant, a field collection might theoretically be maintained for several years without renewal. However, practical experience showed that long-term plantings of highly diverse accessions are fraught with potential problems. There is wide variation in their behavior beyond one or two years in the field; both physical and phytosanitary issues can come into play. On the physical side, large differences in growth habits can mean that very vigorous, highly branched clones out-compete small, low-vigor neighbors to the point of threatening existence of the latter. Large plants tend to lodge, and to become unwieldy to access for observation or management.

Appropriate timing of planting and harvest cycles for clonal renewal helped manage both the physical and biological risks of field conservation. The standard protocol implemented by the late 1970s was that the genebank accessions were planted in overlapping cycles to avoid having vulnerable young plants as the only representative of any given accession. In general, this system involved replanting the collection every year, while leaving each previous planting in the field until it was about 18 months old, thereby giving a 6-month overlap. Based on years of experience, 18 months appeared to be about the maximum time that the accessions could practically be managed at the headquarters’ station without undue risks of losses.

By the late 1990s, an increasing whitefly infestation at the CIAT station resulted in troubling increases in cassava frogskin disease (CFSD) [55]. While risk to all trials was a concern, the germplasm collection was of special priority because of the critical need to minimize losses of any accessions. Despite many years of not knowing the causal agent, relatively effective means of control had been developed, especially the selection of propagative stem pieces from symptomless plants (observation of roots post-harvest, before stakes were cut). Confirmation of pathogen-free planting material could be accomplished by grafting onto highly susceptible indicator plants, usually the variety *Secundina.* However, by 2003, conservation as a field collection was becoming untenable due to widespread CFSD infections. The Cassava Program made the difficult decision, in consultation with the Genetic Resources Program, to discontinue the field-grown collection.

Although the field genebank was discontinued, there was continuing need by various research teams to have access to field-grown plants for various types of trials. As many as 2000 accessions, generated from CFSD-free stock, continued to be multiplied and evaluated for additional years in sites away from the CIAT station, but were no longer considered to be a formal part of conservation of genebank accessions. With diminishing access to planting material, projects aiming to evaluate the germplasm collection tended to focus on the core collection (see later section) or a mini-core subset.

### 6.2. Slow-Growth In Vitro Methods

Two publications from the University of Saskatchewan, a few years after first establishment of CIAT’s genebank, demonstrated successful in vitro cultivation of cassava as rooted plantlets derived from meristems, cultured in sterile media [56,57]. After a brief developmental period to adapt the technology to local conditions, CIAT initiated an in vitro genebank in 1978. As might be expected, there were considerable genotypic effects in response to culture media and growth room conditions. By the early 1980s, the technology and facilities were sufficiently developed to move the entire cassava collection to in vitro culture. While there has been continual fine-tuning of protocols and upgrading of facilities, the key steps developed in the mid- and late 1970s proved to be robust, at least for the cultivated species.

Modifications to slow in vitro growth rates included incorporating growth retardants and managing light and temperature to extend the time required for regeneration. Cassava cultures under slow growth can be stored for an average of a year (varying between 4 and 19 months, depending on the genotype).

In vitro slow-growth conservation of the cassava collection was a landmark success and paved the way for several other technologies and protocols as well, e.g., for pathogen cleaning, rapid multiplication, related work in anther culture for doubled haploid production, mutation, and more recently for transformation and gene editing.

**Figure 6:**
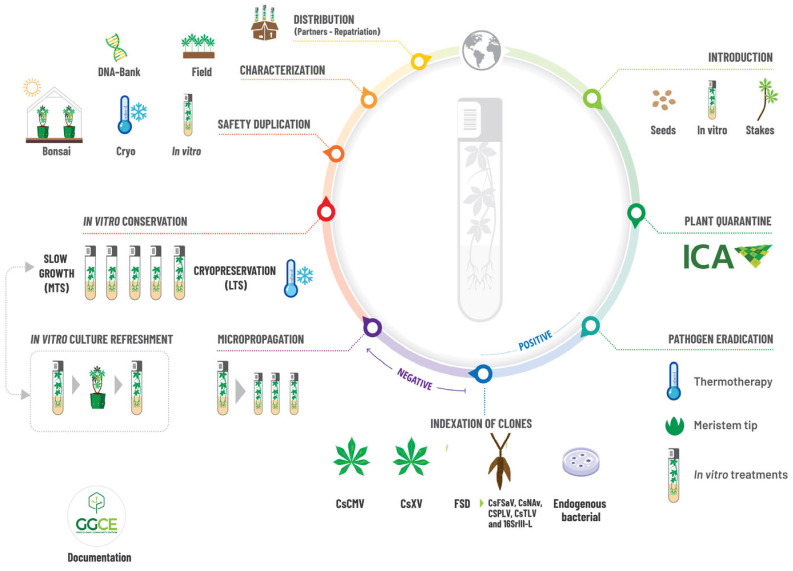
CIAT in vitro conservation laboratory operational scheme for *Manihot* germplasm management.

CIAT produced a comprehensive manual on cassava in vitro conservation management in 2010, representing learning and experiences over almost 30 years [58]. Some fundamental activities are illustrated by a current flow chart (Figure 6) and with additional details in Table S4 [59,60,61,62,63,64]. These include acquisition, pathogen indexing and cleaning, in vitro conservation, and the generation of material for safety backup, characterization, experimental use, and distribution.

In vitro cultures are the only accepted acquisition pathway for clonal materials entering the genebank. Stakes are established under controlled growth chamber conditions for sprouting and thermotherapy treatment. Following surface disinfection, meristem culture (or embryo culture in the case of seeds) is used to introduce the materials to the in vitro collection.

To conserve and distribute the cassava germplasm, quarantine pathogen testing and cleaning through thermotherapy, electrotherapy and/or cryotherapy in vitro treatments are done to comply with phytosanitary regulations established by the Colombian Agricultural Institute (ICA). Screening primarily targets pathogens associated with cassava frogskin disease (five pathogens), cassava common mosaic virus, and cassava X virus.

Each accession is maintained as an in vitro conservation unit consisting of five culture tubes, each containing three plantlets. Four test tubes with NP conservation medium are supplemented with silver nitrate to promote slow growth [60], while one tube is kept on MS medium to ensure availability of actively growing material. The *Manihot* wild relative accessions are maintained in special 12NP medium developed by Velásquez and Mafla [63].

The in vitro cultures are incubated in a conservation room at 23 °C, for a 12 h photoperiod, and at 40/50 µmol·m^−2^·s^−1^ light intensity. Under these conditions, cultures may remain viable for up to two years. Viability is assessed monthly, and plantlets are subcultured to renew the conservation unit in proper medium or produce new material for different purposes such as safety duplicates, characterization, or germplasm distribution. For multiplication, cultures are transferred to propagation media such as MS [59], 4E [61], or 12A3 for wild species accessions [60], depending on the genotype and intended use. Rooting is induced using 17N medium [62] when plants are intended for transferring to soil. Propagation cultures are incubated at 28 °C under a 12 h photoperiod and a light intensity of approximately 80 μmol m^−2^ s^−1^ for 1–2 months before further use.

After nearly four decades of continuous in vitro maintenance, endogenous bacterial contamination and reduced plant vigor have emerged as significant challenges. To address these issues, an in vitro culture refreshment protocol involving temporary transfer to ex vitro conditions, agrochemical treatment, and subsequent reintroduction through meristem culture has recently been developed and is currently being validated for publication. In parallel, droplet-vitrification cryopreservation [64] is being implemented as a long-term conservation strategy to establish both a base collection and safety duplicate copy.

With the inauguration of the Future Seeds genebank complex at CIAT in 2022, the in vitro growth rooms and preparation facilities have been substantially upgraded, now also providing sufficient space for mainstreaming cryopreservation. In September 2023, a partial duplicate of the cassava collection (over 3000 accessions) was transferred to the Future Seeds facility to evaluate plant growth and conservation performance under the new temperature and lighting conditions.

During the first half of 2024, the most critical accessions—those affected by bacterial contamination, low vigor, and/or with limited plantlet availability—were identified and prioritized for further transfer to the new facility. The final relocation of the entire in vitro cassava collection was completed during the second half of 2024. The new facilities for conservation extend over 427 m^2^ versus 175 m^2^ in the old genebank. Additions to space and facilities include a second subculture room and a dedicated sterilization room. The area of the active growth room was increased from 12 to 47 m^2^. Additionally, in 2024, a dedicated cryobank laboratory was constructed to house cryogenic storage tanks for cryopreserved samples. Extra space offers capacity for training and research projects aligned with the innovation and community engagement aims of Future Seeds.

Despite the broad success of in vitro conservation, a major drawback of the system is the delayed access to the accessions for research involving mature plants. Regenerating plants from in vitro culture to provide planting material for field trials is painfully slow. While in theory this should be possible in 8–9 months, the reality is that often around 18–24 months are needed to obtain the required stakes for field trials. This can take even longer for a second level of multiplication for larger trials. After the field genebank was eliminated in 2003, the field testing of accessions became considerably more challenging.

### 6.3. Security Backup Duplicates

A security backup copy for in vitro cultures is also referred to as safety duplication or a black box security system to indicate the protected legal status of the materials. Even if conserved in the facilities of a different organization, the cultures legally remain under the control of CIAT. They can only be returned to CIAT for the purpose of replacing lost materials, or, alternatively, are destroyed. The system began as a collaboration between CIAT and the International Potato Center (CIP) in Lima, Peru, based on an agreement signed in 2005. The centers collaborated to duplicate each other’s collections of cassava and sweetpotatoes, i.e., CIAT held an inactive copy of CIP’s sweetpotato collection and CIP held an inactive copy of CIAT’s cassava collection. Refreshed material was sent approximately every 12–18 months from the base curating center to the security backup center. According to Genesys records, the most recent batch of 600 cassava accessions was sent to CIP in 2023. Since the establishment of the backup agreement with CIP, CIAT has sent 35,692 samples representing 5744 accessions. Wild species accessions were initially considered to be part of the backup agreement; however, restrictions imposed by Peru’s plant health authority (SENASA) limited the import permit exclusively to *M. esculenta*.

In 2018, CIAT and AGROSAVIA of Colombia established an alternative security backup at the La Selva research center in Rionegro, Antioquia. Apart from the convenience of dealing only with internal Colombian quarantine measures (rather than international regulations between Colombia and Peru), this backup system allowed for duplication of some of the wild species. After conducting visits and technical adjustments to install and operate growth chambers in AGROSAVIA, the first batch of cultures was shipped to the La Selva facilities in October 2023.

The COVID-19 pandemic during 2021 and 2022 created substantial challenges and disruptions to cassava in vitro conservation at CIAT and led to a die-off of most safety duplicated accessions at CIP. However, rather than re-initiating the safety duplicate at CIP, the genebank focused on strengthening the AGROSAVIA-based system.

### 6.4. Critical Challenges and Successes in the Search for High-Efficacy Cryopreservation

Cryopreservation is the storage of germplasm at ultra-low temperatures (−196 °C) using cryogenic media, typically liquid nitrogen. This temperature effectively stops the biological activity of the plant cells and nearly eliminates the need to regularly subculture the plants in vitro. Panis et al. [65] list some of the advantages and disadvantages for cryopreservation:

Advantages:

- Minimum space requirements;
- Low long-term maintenance costs;
- High genetic stability;
- Long-term storage (>100 years);
- Largely independent of stability of electrical supply (added by the authors of the current report).

Disadvantages:

- Restricted availability;
- Restricted accessibility;
- Access to liquid nitrogen is limited in some situations;
- Need for specific protocol development;
- High initial workload and costs to cryopreserve clonal plants.

CIAT pioneered research on cassava cryo in 1985 and has given varying degrees of research emphasis to improving the techniques since that time. Cryopreservation research has been managed for many years under the leadership of CIAT’s Biotechnology Research Unit (BRU. In the future, cryopreservation of the cassava collection will be mainstreamed as part of the genebank operations in CIAT’s Future Seeds facilities. Several procedures have been developed that involve the extraction of freezable water from the tissue cells before rapid cooling. If the cell interior is sufficiently dehydrated, it undergoes vitrification, which is direct transition from the liquid phase into an amorphous phase (or glassy state), while avoiding the formation of lethal crystalline ice. A modification of this technique, called droplet-vitrification, further reduces the chance for ice crystal formation. It achieves this through the application of ultra-fast cooling and rewarming rates, using aluminum foil and small amounts of cryoprotectant solutions. The technique has now been developed for different crops, including cassava. The droplet-vitrification methodology appears to be technically mature [64], to be implemented as a long-term conservation and safety backup method. The Future Seeds facility at CIAT has a laboratory and cryobank area in place and is implementing the technique. Initially, in a pilot phase, cryo could be considered a backup system, but eventually would likely become the base collection. Some poorly understood risks remain. These include long-term mutation from background ionizing radiation, which could suggest the need for some periodic recovery, testing and renovation as part of best practices for cryopreservation.

At CIP, CIAT and IITA, 30% minimum plant regrowth (the successful development and regeneration of the whole plant) is used as a threshold for potato, yam and cassava, and 20% for sweetpotato. After more than 30 years, and with only sporadic and low-level funding for the most part, CIAT has refined the technology to a high level of success for nearly all tested genotypes. In tests of 1466 clones, including the most representative collections from Brazil, Colombia, Peru, Venezuela, and other countries, as well as some improved hybrids, only 2–3% showed a response of under 30% recovery with the droplet-vitrification protocol (R. Escobar, pers. comm.)

Escobar [66] suggested that a cryopreservation unit should consist of 100 meristems of each target accession, distributed in cryovials of 10 meristems each. In addition, each vial should include a sentinel clone with a known high post-freezing response (such as BRA 856) as a monitor for the system.

Protocols have also been adjusted for wild *Manihot* species, some of which behave very poorly in vitro or even in the field, making their successful conservation very challenging. Plants have been recovered from cryopreservation for *M. esculenta* subsp. *flabellifolia, M. esculenta* subsp. *peruviana* and *M. carthaginensis*.

An international feasibility study of independent experts proposed a global cryovault for conserving vegetatively propagated species, along the line of Svalbard for seed conservation [67]. Nearly a decade later, development of regional cryohubs for clonal and recalcitrant crops is now in progress—one in the Americas led by CIP, one in Africa led by IITA and a third in Europe led by the Alliance of Bioversity and CIAT. The CIP cryovault was officially inaugurated in Lima, Peru, in November 2025, and is expected to include the CIAT cassava collection (https://cipotato.org/cipatplanttreaty/regional-cryohubs-recalcitrant-crops/ (accessed 14 March 2026)).

### 6.5. The Bonsai Conservation System

After elimination of the field genebank, just a single in vitro collection in one site would have implied an unacceptable level of risk. The 2002 CIAT Genetic Resources Annual Report [68] notes: “As a result of the cleaning and indexing effort launched in 1996, 73% of the cassava collection is now indexed against viruses of quarantine importance and available for distribution. As a by-product of this work 2169 clones have been established as a bonsai collection that may result in a low-cost alternative to the field genebank, before the work on the cryo-collection is completed.”

The colloquially named bonsai conservation system embodies the concept of producing cassava plants with moderately restricted growth by limiting container size, plant nutrients and water, along with strategic pruning. It is designed to offer space efficiency, resource conservation, phytosanitary control, and accessibility, compared to field collection.

To serve as a valid conservation system, these plants should ideally be grown under highly controlled, insect-free and limited-access conditions in a greenhouse. Initially, there was considerable experimentation on the growth conditions that would achieve the best balance between slow growth (minimize space used and extended regeneration times) and multiplication rates (number and quality of propagules that could be obtained to produce material for field trials). From this perspective, priority for the bonsai collection would be for materials most used/requested by CIAT research programs. There was an informal discussion about the possibility of ICA certification of phytosanitary status of the bonsai plants, especially to qualify for international shipments. However, this was in fact not pursued vigorously and did not materialize due to FAO recommendations for the safe international movement of cassava only as in vitro cultures [38]. Nevertheless, the bonsai collection has served as a valuable local backup, enabling the recovery of accessions that had lost viability or vigor due to contamination or poor response under in vitro conditions.

Niño-Jimenez et al. [69] describe research to refine the bonsai system, working with conservation system variables to minimize the loss of material. Modification of the substrate, use of a biological inoculant, moist chamber conditions, fertilization and controlled pruning allowed the establishment of accessions in plastic containers with a loss rate of less than 4%. Maintaining plants for more than three years (and even cases of more than five years) is possible and comparable to or better than what is achieved with in vitro techniques. As of 2025, the bonsai collection included 5749 accessions, each represented by two potted plants: one maintained in a greenhouse and the other in a screenhouse. This system is not yet reported from other cassava germplasm collections.

### 6.6. True Seeds to Conserve Allele Diversity

CIAT does not currently conserve true seeds as part of its cassava germplasm conservation system. Although cassava ex situ collections appear to have been conserved around the world exclusively as clonal repositories (field, greenhouse or in vitro), hypothetically, conservation of the full genetic diversity of the species as true seeds is also possible. For the development of new varieties, breeders are interested in sources of specific alleles, and those sources do not need to be specific genotypes (or varieties). However, there are no standardized recommendations for genebanks consisting of true cassava seeds. There would be several advantages to this approach, but also some challenges. Although fully controlled experiments for long-term storage have not been done, IITA reports storing seeds (of approximately 6–8 per cent moisture content at 5 °C and 60% RH) with no difference in seed viability between the ages of 0 and 7 years [70]. Seeds can also be stored in liquid nitrogen, presumably for a much longer period, and therefore potentially with less complexity in managing a regeneration system [71,72].

Because cassava landraces are all highly heterozygous, their progeny—whether from self- or cross-pollination—will segregate widely. Reproducing the parent genotype through seeds is not currently possible. However, seeds can be used to conserve the full array of the alleles in each accession, if the appropriate pollination and sampling methods are used. A Crop Trust working group on cassava conservation strategies [8] suggested that the best way to develop seed populations for genetic resources conservation purposes would be by selfing plants within each landrace accession. This technique has the advantage that only the alleles from that specific accession are passed on to progeny through the seed, as opposed to the mixing or dilution that would be caused by crossing with other accessions (or if open-pollinated seeds were collected). A disadvantage of selfing is that cassava often suffers moderate-to-severe inbreeding depression. The plants that result from self-pollination will generally be less vigorous than the parent. This loss of vigor can compromise their ability to continue to produce seeds, or even to reproduce vegetatively.

Another challenge is that obtaining seeds from cassava, especially from controlled pollinations, is a somewhat tedious and costly process. Significantly, there is currently not a field collection at CIAT. Further, the ability to produce seeds varies widely among accessions—from profuse seed producers to clones that produce no seeds under most conditions. However, research and continuing practical experience show that pruning and extended daylength through supplementary red lighting can improve flowering in cassava [73,74].

As gene tagging becomes more precise, faster, and more economical, the best parents can be identified regardless of whether they are derived from a seed or are original landrace varieties. Apart from being an effective long-term conservation system, especially in a cryopreserved state, seeds are an easier way to exchange genetic resources internationally.

### 6.7. DNA, Tissue Samples, and Herbarium Specimens

CIAT stores DNA from about 4000 cassava accessions at −80 °C in an ultra-low-temperature freezer. Samples have been used in various genotyping studies, most notably [10]. Sample curators anticipate that the DNA should retain high quality for genotyping analysis for 10–15 years.

The genebank preserved lyophilized leaf tissue beginning in 2017, as a source for the DNA extraction described above. There are approximately 3700 accessions conserved in this form. High-quality DNA can still be extracted from the earliest tissue, suggesting long-term stability. Databases for both DNA and tissue samples are managed internally within the CIAT Genetic Resources Program.

The CIAT herbarium, established in 1978 and registered with the National Registry of Biological Collections of the Alexander von Humboldt Institute (http://rnc.humboldt.org.co/admin/index.php/registros/detail/2686), serves as the botanical reference collection for *Manihot* spp., common bean (*Phaseolus* spp.), and tropical forages conserved at the Future Seeds genebank. Its main roles are taxonomic verification, correction of labeling errors, and resolution of segregation or cross-contamination issues in genebank accessions. The collection includes 153 *Manihot* specimens representing 42 species donated by CENARGEN/Embrapa (Brazil) and an additional seven species from Mexico. There is no overlap among the species contributed by the two countries (Figure 5 and Table S5). The potential overlap of genetic identity among in vitro accessions and herbarium specimens has not been investigated.

### 6.8. Special Conservation Challenges and Solutions for the Wild Manihot Species

The wild *Manihot* species are notoriously difficult to maintain ex situ. In nature, they are mostly perennial, and all appear to be cross-pollinating and to reproduce primarily by seed. The main efforts in conservation have been by Embrapa-CNPMF in Cruz das Almas, Bahia; CENARGEN in Brasilia; the Federal University of Brasilia (Dr Nagib Nassar); IITA in Ibadan, Nigeria; and CIAT in Colombia.

Much of the reporting on early work with wild species conservation at CIAT resides in unpublished internal documents, such as annual reports, conference proceedings or newsletters. Fortunately, many of these documents have become more available and more easily accessed in recent years, especially through the CGIAR centralized repository of documents (https://cgspace.cgiar.org/home).

In the first years after their introduction, CIAT grew some of the wild species in the field on approximately a 2-year renovation cycle. Given the broad ecological range of adaptation of the *Manihot* species, it is difficult to mimic their necessary edapho-climatic conditions for propagation. The CIAT station is perhaps especially poorly suited to many of the species, which generally originate in lowland high-temperature conditions, in poor soils on forest margins, and often dry environments. Many are adapted to somewhat acid soil conditions, in contrast to CIAT’s more pH-neutral or alkaline-tending soils. A different pest and disease complex is present at CIAT compared to most of their native habitats. Plants often grow poorly, do not produce seeds, and stem cuttings do not sprout well.

Suggestions for the mitigation of the challenges include seeking out sites with more suitable edapho-climatic conditions specific to the wild species’ needs. In the end, this was not possible with available funding. For at least the past 30 years, only a very few wild species have been used in field experiments. However, the Breeding and Genetics sections of the Cassava Program carried out modest evaluations and crossing experiments.

Chavez et al. [75] reported on priorities for the development of techniques for the conservation and use of wild *Manihot* spp.:

- Develop methods for breaking dormancy and overcoming seed sterility.
- Develop techniques for embryo rescue, for seeds that germinated poorly with conventional germination procedures, and for their subsequent in vitro culture.
- Develop methods for in vitro conservation from vegetative tissue of wild *Manihot*.
- Study the cross-compatibility relationships (interspecific crosses between wild species and cultivated cassava).

Given what we know now about the challenges in each one of these objectives, this was a very ambitious set of ideas for a modest project. Nonetheless, this important early progress set the stage for later successes in maintaining up to 33 wild species in the genebank (including the two *M. esculenta* wild sub-species *flabellifolia* and *peruviana)*.

Accessions are currently held as in vitro cultures (Table S3), and many of these are being established as part of the security backup system with AGROSAVIA in Colombia. Many are also duplicated as bonsai plants in a greenhouse at CIAT. Seed conservation for long-term storage, with a pool of seeds that represents the alleles of a population, and based on on-going reliable seed production, remains a goal yet to accomplished.

### 6.9. Genetic Stability In Vitro

In addition to the capacity for continual secure regeneration over many subculturing generations (in perpetuity conservation), long-term genetic integrity is also fundamental to an effective in vitro system. This parameter has been a concern addressed from the very early days of establishment of the in vitro genebank, with increasingly sophisticated tools over time. Early trials compared accessions kept continually in the field with those that progressed through multiple cycles of in vitro culture, with no evident morphological differences. Angel et al. [76] were the first to test whether longer-term in vitro storage of cassava germplasm (10 years) caused genetic changes detectable at the DNA level, based on RFLPs and RAPDs. No DNA rearrangements or polymorphisms were detected in plants retrieved from in vitro storage. They concluded that cassava germplasm can be safely conserved in vitro for long periods without compromising genetic integrity. Mafla et al. [77] evaluated the genetic stability of six accessions after 20 months of slow-growth storage in medium supplemented with silver nitrate. The evaluations—morphological (20 descriptors), biochemical (profiles of 15 isozyme systems involving 20 genes) and molecular (AFLP fingerprints)—confirmed genetic stability of in vitro plants over this relatively short time frame. Nonetheless, molecular tools have advanced logarithmically since the mid-1990s, and a renewed look at in vitro genetic stability is warranted.

### 6.10. Genetic Diversity Lost over Time

Safekeeping of a collection implies an obligation to monitor and document losses, assess the reasons for those losses, and recommend steps for improvement. Losses of accessions, at some level, are a normal part of what nearly every genebank experiences. In general, clonal collections represent greater levels of risk for loss—brought about by factors such as: (1) greater exposure to variables in the external environment (field); (2) the need for more frequent renewal compared to seeds in cold storage (field and in vitro); (3) greater risks of pathogen infection and transfer via cloning (field and in vitro); (4) an artificial growth environment (in vitro) that is not uniformly suitable for all accessions; or (5) sensitivity to disruptions in laboratory security and stability.

Table 6 shows estimated cassava accession losses since their year of original collection, from each country of origin. Not much detail is available for the causes of losses for specific groups of materials. Where rather large losses are noted—especially Colombia, Ecuador, Panama, Peru and Puerto Rico—disease issues during the quarantine phase of introduction appear to be the most prevalent problem. These were usually situations where disease symptoms were detected in the collected material after introduction into the quarantine process. The normal and appropriate response by quarantine officials was to destroy infected material. Introductions into quarantine were often formally registered into the genebank and assigned accession codes prior to actual pest or pathogen assessment, but in fact some of these accessions never arrived at the CIAT facilities in Palmira.

Other significant losses occurred from variable and unsuitable soil conditions, especially highly saline areas. The CIAT headquarters station suffered initially from sectors of soil with high salinity. Within a few years after establishment of the germplasm collection (by the mid-1970s), managing this problem became better understood, by simply planting only in areas without identified salinity problems.

After the post-quarantine field establishment, complete accession losses from pests and diseases seem to have been low. The field collection was consistently monitored on a regular basis, specifically to identify potential pest and disease issues. If risk levels for any problem rose to a threshold level, control measures were taken to prevent losses [54].

In vitro losses have been due to the convergence of several likely factors. These included bacterial or fungal contamination, loss of vigor due to environmental stress caused by infrastructure adjustments in 2011, low response of some accessions to in vitro treatments, and poor rooting, especially for wild species. Recent annual reports of the Genetic Resources Program also highlight management challenges that contributed to in vitro losses. Notably, there was a subculturing backlog and some loss of accessions during the lockdowns of the COVID-19 pandemic in 2021 and amid the subsequent civil unrest in Colombia. There was limited access to the in vitro collection, which exacerbated contamination with both endophytic and other bacteria, in addition the loss of plant vigor due to aging, causing some continuing losses.

Losses among wild *Manihot* relatives have been strongly associated with the highly divergent adaptation of the different species to all forms of conservation (field, potted plants, in vitro). For example, there is an acute challenge of a lack of continued root production under in vitro conditions. Consequently, the level of losses is closely associated with species identity. Perhaps not surprisingly, the most successful have been accessions of the two *M. esculenta* subspecies, *flabellifolia* and *peruviana*.

In 2011, CIAT reported 883 accessions of 33 wild species or subspecies in vitro (Genesys.org historical records) (while *flabellifolia* and *peruviana* are subspecies of *M. esculenta*, they are found only in the wild, and therefore are listed here with the wild species rather than cultivated cassava, *M. esculenta*). However, following genebank renovations, and the collection’s temporary relocation in 2011, there was a progressive decline in root production of wild species in vitro and increasing bacterial contamination. Since the field collection had already been eliminated, there was no copy available as a backup to replace accessions lost in vitro. The current 377 accessions (Table 5) from 22 species and two subspecies represent a loss of almost 52% compared to 2011 pre-relocation numbers.

Since 2021, CIAT began to categorize accessions for risk of loss and causes of loss in vitro. The primary indicators include the progressive decline in explant viability and physiological vigor, microbial contamination, and recalcitrance to in vitro conditions, especially in wild species. Transfer of the in vitro genebank to the new Future Seeds facility in 2024–25 was also demanding of resources and placed stress on conservation integrity.

Given this history of losses—with about 25% of cassava accessions lost since their original introduction—what measures were taken or should be taken to recover any lost genetic diversity? The principal attempts at recovering lost material are reported for Peru, both through collection expeditions [27] and re-introduction from the Peruvian national cassava genebank. Unfortunately, the materials re-collected by Salick et al. did not successfully pass through ICA quarantine in Bogota, and were ultimately not included in the CIAT collection. Collection expeditions are complex and costly, so funding is challenging for re-collection, except in extreme conditions of loss, or where there is strong evidence that alleles of high value could be retrieved. In comparison with the need to collect in areas not previously explored, re-collection in areas where only low or moderate loss occurred will probably continue to have a low priority.

Providing greater future security includes managing contamination, staying current on subculturing, and further research on the diverse culture demands of the wild species. The addition of a fully functional safety backup strategy, and future implementation of a cryo-collection, will help reduce losses to very low and acceptable levels. The parallel bonsai conservation scheme (potted plants), including wild species, and the security backup arrangement with AGROSAVIA in La Selva facilities in Colombia further contribute to reduced threats of losses.

### 6.11. Managing the Error Rate in Accession Identification

An error in accession identification is generally not as serious as complete loss of an accession, i.e., the genetic value of the material remains the same for possible future use. However, such errors do decrease the value of an accession by potentially introducing confusion and follow-up errors of many types. In general, identification errors generated prior to complete bank genotyping [10] cannot readily be corrected, given the lack of a field collection as a reference for true-to-type confirmations. Typically, these errors are only found if a well-known accession does not look like it is supposed to look, in the eyes of the professionals (often the plant breeders) after a period of regeneration either from the field or the in vitro collection. Since the environment can cause many phenotypic variations in cassava, generally only very distinctive variations in the more stable traits, such as those used for characterization (see Table S6), will support an error diagnosis [6]. That said, there have been multiple examples, based on the authors’ personal experiences, of errors identified in this way. Sometimes alternative sources to recover the original genotype are available, and sometimes they are not.

Germplasm conservation, especially of an annual, clonally propagated crop, has many steps in every renovation and conservation phase, each with the potential for the introduction of human error in accession identification. Hypothetically, the error rate for an annually renewed clonal collection might be about ten or more times that of a seed-based collection of orthodox seeds, where regeneration takes place only very infrequently.

With the field collection, for about the first decade after establishment, these multiple steps usually meant handwriting a field or greenhouse label, hand-entering IDs in field or lab notebooks, and then the typing of those notes by a secretary or specialized data transcriber for archiving and reporting.

Each of the following steps (among others) during the years of extensive manual information management allowed the introduction of potential human errors:

During collection and introduction:

- Mistakenly collecting more than one clone as part of a variety designation and assigning them to a single accession number.
- Potential for misinterpretation or misunderstanding between collector and farmer/donor of a variety name or its characteristics. This may be especially acute in the case of indigenous languages.
- At the time of collection, labeling of stem bundles.
- Labeling of plants during quarantine.
- Developing laboratory notes during quarantine.
- Re-labeling of stem bundles for shipment to the CIAT experiment station.
- Updating accession inventories by handwriting, typing or another manual entry form.
- Developing the field book to include new introductions to the field collection.

During field conservation:

- Labeling of stem bundles from each plot at harvest.
- Chance of lost labels during transportation, treatment, replanting.
- Developing the field book for annual regeneration.
- Typing inventory for annual reporting and archiving purposes.

During in vitro conservation:

- Initial culturing from the field collection:
  ○ Labeling of stem pieces in the field.
  ○ Labeling of pots in the greenhouse to produce shoots for meristem culture and registry in the lab notebook.
  ○ Labeling of containers during processing of shoot tips, and meristem extraction and registry in the lab notebook.
  ○ Labeling of test tubes for slow-growth culture and registry in the lab notebook.
  ○ Typing or digitizing any of the above information.

- During periodic regeneration (1 to 2 years):
  ○ Labeling of containers during subculturing processing and registry in lab notebook.
  ○ Labeling of new test tubes to begin new grown cycle and registry in lab notebooks.
  ○ Typing or digitizing any of the above information.

Estimation of past or current error rates is difficult but, at both the field and laboratory levels, there was continual attention to improving systems to reduce error rates. Most of these issues have been managed by moving toward barcode-enabled tracking and may further be improved in the future by RFID tags to ensure sample integrity in vitro. An effective documentation system is fundamental for ensuring data integrity, traceability, secure storage, and controlled access to information associated with each accession (see later section).

## 7. Characterization and Duplicate Identification

The classical terminology for describing genebank accessions has been characterization and evaluation. Although molecular data are not easily observable traits in the traditional sense, FAO and CGIAR genebank practice now treat genetic marker profiles as an integral extension of characterization. Grower and consumer knowledge (or cultural information) is generally considered a part of passport data, but was usually de-prioritized in early collections. Now, there is greater appreciation of that knowledge.

Long before genebanks were established, cassava farmers certainly recognized a broad array of color, shape, size and behavior traits that allowed them to distinguish among their varieties. Following from this knowledge, there also often evolved a perceived association between these easily observed traits and traits of importance in productivity, resistance, quality and others. For example, farmers might associate purple petiole color with good cooking quality if their best-tasting varieties consistently had purple petioles. Farmers and processors sometimes associate high cyanogenic potential with good starch quality [24]. Such associations may be strongly held but should be examined based on systematic research evidence.

While characterization and evaluation historically constitute two distinct ways of describing the genetic diversity within collections, in fact there is a continuum between the two categories—from highly heritable to low heritability, and from traits that appear to have no economic value for users to those that are of fundamental importance.

### 7.1. Characterization Based on Morphological Traits

Cassava breeders and genebank managers have generally come to rely on basically the same key traits as farmers for distinguishing plants in the field. Although CIAT developed a basic set of characterization criteria soon after the establishment of the field collection in the early 1970s, the formal descriptor list for cassava evolved modestly over time. IBPGR convened a group of experts in 1983 to formalize a recommended list. In 1995, cassava specialists from seven countries met in Cruz das Almas, Bahia, Brazil and updated the list, resulting in the publication by Fukuda and Guevara [5]. Fukuda et al. [6] compiled information that had evolved over time, jointly and in parallel, between Embrapa/CNPMF in Brazil and CIAT, and for the first time integrating input from Africa (Table S6).

Some of these descriptors do not fully meet the criteria for being stable, i.e., with little environmental influence on their expression. Several of the traits suggested for scoring at harvest are clearly quantitative in nature, and in fact very influenced by environmental conditions. These include yield and yield components, root quality traits like starch or dry matter content, cyanogenic potential, and post-harvest deterioration. It is not quite clear why these traits are on the descriptor list; in standard practice, they should be viewed more as criteria for evaluation. The CIAT collection has been fully characterized, and these data are available in Genesys.

Cassava has never had the benefit of extensive morphological marker gene studies. Hershey and Ocampo [78] described eleven tentatively simply inherited traits, some of which became part of the descriptor package (Table S7) [78,79,80]. However, there is currently little interest in further exploring the classical genetics of crop descriptors since there is now the easy possibility of many thousands of DNA-based markers.

While morphological descriptors are still useful for a quick field comparison of materials, or to describe new varieties being released to farmers, they have never provided the level of confidence needed to confirm duplicate accessions. Even where the characterization criteria for two clones fully match, this has not been considered sufficient evidence of clonality. Nonetheless, even with the use of molecular-based description, there is still the broad expectation that the verification of variety identity should include field-based morphological descriptors.

### 7.2. Revelations from Local Variety Names

Farmers almost always assign names to their landrace varieties. The integrity of these names within a household and community is critical for purposes of associating varieties with their uses in the production and food systems. Names often indicate:

- An identifying plant trait—especially shapes or colors of plant parts such as leaves, stems or roots.
- Origin (e.g., *Caucana*, *Llanera*, *Venezolana*, or *Palmireña*), especially if introduced from outside the farm or village.
- An honor to a respected person or the donor of the variety.
- Special features related to use or taste, such as exceptional sweetness or bitterness.
- A growth trait such as earliness, branching habit or height.

Of the 4406 landrace accessions that were registered with common names in 2023, 72% of those names are unique. However, names that were perhaps identical at the farmer level may have minor spelling variations in the genebank registry. This is probably true for many names, but especially for those in a local language not familiar to the collectors. An extreme example seems to be from a collection expedition in the Pasco province of Peru by Jan Salick and colleagues in 1986 (www.genesys-pgr.org/c/cassava (accessed on 23 April 2025)). Seven accessions were given the following names by the collectors: *Yapam*, *Ya’Pam*, *Yapamam*, *Ya’Pamam*, *Yapaman*, *Yapamon*, and *Yapamuen*. It is reasonable to hypothesize that some of these different spellings could be the result of slightly different ways that individual collectors heard the pronunciation of a given name.

Some of the most common local names across regions (English translations and number of repetitions in parentheses) are as follows:

- *Algodona* (cottony) (27);
- *Amarilla*, *La Amarilla*, *Yuca Amarilla* (yellow) or *Yema de Huevo* (egg yolk) (68);
- *Blanca*, *La Blanca* or *Yuca Blanca* (white) (150);
- *Brava* or *Yuca Brava* (bitter) (26).

Interestingly, names rarely appear to derive from traits related to pest or disease resistance, or adaptation to soil, climate or cultivation practices. This lends some support to the hypothesis that nearly all successful varieties will necessarily have basic adaptation to these biotic and abiotic conditions within the production environment where they were grown. Names would therefore not be determinative in distinguishing among varieties.

Some of the descriptive names assigned by farmers can certainly provide breeders with a clue about possible traits of interest for breeding. Names related to early maturity (*Seismesina* (six months); *Sietemesina* (seven months); or *Temprana*, *Tempranura*, *Tempranita* (early)) should be good candidates to prioritize for the evaluation of root bulking patterns. *Yema de Huevo* and *Yuca Amarilla* will have yellow root flesh and should be candidates for high carotenoid content for biofortification breeding.

Apart from giving due regard to the donor-provided name, the genebank curator and breeder should rely on other criteria, especially thorough phenotyping, to confirm traits. Additionally, names have little value in the identification of duplicates—either to suggest that same names indicate duplicates or that different names necessarily indicate genetically distinct clones.

### 7.3. Characterization Based on Gene Products

Molecular tools evolved quickly, beginning about a decade after the establishment of the genebank, and have moved to the forefront of diversity analysis and duplicate identification. These tools are broadly grouped into those that measure differences in biochemical markers (gene products), and those that measure DNA sequence variations (Table S8). Some recent comprehensive reviews provide a more complete background, with hundreds of references for possible follow-up, for example, [4,10,81,82,83,84,85,86,87,88,89,90].

#### 7.3.1. Isozymes

Isozymes are enzymes that have the same basic biological function but differ slightly in their amino acid sequences. Isozyme analysis was used already in the 1950s as one of the earliest molecular tools for assessing genetic differences. CIAT initiated cassava isozyme research in the 1980s based on the αβ-esterase system. At the time, it was perceived as a powerful advance toward precision variety identification and diversity analysis, and phylogeny and evolution studies. To function in these roles as markers, isozymes should be:

- Polymorphic—They should show variation among different cassava accessions.
- Heritable—They should be passed on from parent to offspring without being affected by environmental factors.
- Stable—They should be detected reliably and consistently in different tissues and stages of development.
- Independent—They should not influence each other or other traits of interest.

CIAT identified 12 isozyme systems with a good fit to these criteria [81]. These isozyme patterns are published as part of the cassava database available in Genesys.

Chavarriaga et al. [82] reported on a study of the core collection, where a total of 57 alleles were detected among the 12 isozyme systems, with an average of 4.75 alleles per system and a range of two to nine. The most polymorphic systems were EST, PGM, and PRX, which together accounted for 47% of the total variation. The least polymorphic systems were ACP, AAT, G6PDH, and TPI, which accounted for only 7% of the total variation.

#### 7.3.2. Metabolomics

In the mid-2010s, CIAT began to tap into the promising results of metabolomics being used by the Paul Frazer laboratory at the Royal Holloway University of London [83]. These analyses were based on the generation of pan-metabolomes for root and leaf tissues to enable the identification of metabolic sectors underlying traits of interest. For example, tolerance to whiteflies was linked to cell-wall-related phenylpropanoids or apocarotenoids in the leaves. In general, for the leaf metabolome, there was a clear correlation with genotypic data, as would be hoped for in a diversity analysis. However, the analysis of the root metabolome did not reveal an association with adaptive or agronomic traits. The authors suggest that a greater in-depth study of the root metabolome could be important for linking selection criteria with consumer-preferred quality traits. In terms of genebank management, metabolomes appear to be somewhat less definitive for characterization, compared to DNA markers.

### 7.4. Characterization Based on DNA Markers

Genebanks are increasingly exploiting the rapidly evolving capacity to look more precisely at variations in DNA sequences among accessions. All environmental influences are eliminated, and the number of variants is potentially in the hundreds of thousands. DNA-based markers are useful, inter alia, for the following:

- Identifying duplicates;
- Determining genetic distances between individuals or populations;
- Taxonomy;
- Developing hypotheses about evolution, domestication and early distribution;
- Measuring genetic integrity under long-term storage conditions (slow-growth in vitro or cryopreservation);
- Correlating traits of interest with DNA variations to facilitate selection (genome-wide association analysis);
- Identifying heterotic groups to maximize genetic gain.

A detailed history of cassava genomic characterization is provided at https://phytozome-next.jgi.doe.gov/info/Mesculenta_v8_1.

### 7.5. Marker-Based Duplicate Identification and Implications for Accession Management

From the first days of the establishment of the cassava germplasm collection, genebank managers understood that there would be duplicates among collected materials. The appearance of duplicates has straightforward explanations in most cases. Collectors working across many farms could not possibly be discriminating enough to recognize or to make judgements about collected varieties that may look very similar but could either be actual clonal duplicates or simply be very similar in morphological traits. Even within a given field, two plants of the same clone can look rather different, and farmers themselves might not distinguish varieties with accuracy. Nominal duplication could occur from labeling/tracking errors in field and/or in vitro conservation systems during decades of conservation efforts based on handwritten labels (as noted in the earlier section). In these cases, the same code could be assigned even to very distinct accessions. The extent of either type of error is not currently quantified.

Based on review of passport and characterization data, and field observations, Hershey [8] estimated that 10–20% of CIAT’s collection could be duplicated accessions. Duplicates matter on several levels: they add costs to conservation and evaluation, introduce errors into estimates of genetic diversity, and increase the risks of introducing unwanted inbreeding into breeding systems.

At the farm level, complete or true duplicates (all base pairs on all chromosomes identical) come about in only one way: they derive from a single seed at some historical point in time, followed by generations of clonal propagation. However, the inverse is not always true: not every plant derived from a single seed will be a complete duplicate of the others. There may be the introduction of somatic mutations over multiple clonal propagation cycles to create sub-clones. This is well-documented in clonally propagated crops like potato, especially in older varieties [91,92].

The technology for confirming clonal duplicates has developed slowly. Methods that rely on passport data or characterization simply do not, by themselves, confidently identify duplicates. None of the analyses applied up to 2024 had come close to providing the required level of confidence for elimination of duplicates broadly across the genebank accessions.

Carvajal et al. [10] analyzed 5302 accessions in CIAT’s cassava collection with SNP and SilicoDArT markers. Based on these marker patterns, about 52% of accessions showed possible redundancy (or duplication), with a range from 2 to 87 accessions within clusters, while a total of 1567 (29.6% of total) accessions had no redundant accessions. Based on these analyses, the likely minimum number of unique accessions in the collection (from both redundant and non-redundant clusters) would be 2518. However, exact redundancy remains to be confirmed with higher density markers, ideally combined with field and laboratory comparisons of the most stable descriptors. This basic information will support a fully rational system for conservation, evaluation and use.

Table 7 shows data from 12 of the larger SNP/SilicoDArT redundant groups (from 20 to 86 landrace accessions per group). Some redundant varieties have broad geographical dispersal while others have remained more local. Groups with large numbers of tentative duplicates may be especially informative. Identical accessions (or differing only in a limited array of somaclonal variations) that have been collected across broad geographical areas were probably passed down over many generations because of their exceptional qualities for growers and consumers and were traded across regions.

SNP/SilicoDArT group 127/3139 is especially notable for the fact that its varietal components were collected across 12 countries—broadly throughout the Americas, as well as in Thailand, Indonesia and Fiji. Widespread distribution of specific varieties (or closely related varieties) offers both opportunities and caution to breeders. First, their value within the genebank seems obvious, in terms of a genotype having broad grower and consumer appeal (although phenotypic performance is not necessarily an indicator of breeding value). On the other hand, breeders need to be very aware of the possibility of the deleterious effects of inbreeding if crossing different accessions that are in fact genetic duplicates or near-duplicates.

In contrast to group 127/3139, four groups—1182/4747, 2443/4531, 2729/3639 and 4535/4313—are large groups of almost exclusively Colombian origin. While these landraces are highly appreciated locally, either they were not extensively introduced to other countries, or they may have been introduced but failed to gain farmer adoption.

There appear to be some regional differences in levels of redundancy (or uniqueness). Across eastern and western South America, the rates of potential duplication are rather similar: nearly 30% of accessions are unique in both areas. On the other hand, only 16% of accessions collected from Meso-America and the Caribbean are unique. This may be due to cassava’s likely origin in South America, and the tradition of developing new varieties from seedlings, which is reported to be more prevalent near the center of origin [29]. Ongoing comparative studies between CIAT and IITA genebank data will provide insights into possible examples of historical movement of varieties from the Americas to Africa as well.

## 8. Documentation

An accurate and secure documentation system is fundamental for the effective conservation and use of the cassava collection. It ensures data integrity, traceability, secure storage, and controlled access to information associated with each accession. A robust information management system supports recording routine operational activities related to standardized quality management of a collection conserved using different methods (e.g., in vitro, greenhouse, and safety duplication) and accommodates evolving technical and regulatory requirements.

The early field collection (late 1960s/early 1970s) began with handwritten field notes, which were sometimes typed up by office staff for filing. The collection documentation system largely followed the evolution of tools and systems used by the CIAT cassava breeding section. By the 1980s, with establishment of an in vitro system, field and in vitro data merged into a unified in-house Oracle database.

For several years, the in vitro collection was managed using an Oracle V.7.0 database, which recorded basic passport information, distribution records and micropropagation events corresponding to the generation of new in vitro conserved units for each accession. The system included integration with barcode-based identification and mobile data capture tools. In 2016, a customized version of the GRIN-Global database was implemented, expanding the scope of information stored and enabling more detailed tracking of operational activities. The system enabled comprehensive storage of the passport, taxonomic, characterization, and evaluation data available for each accession. The stronger inventory management system of this database supported tracking of accession movements during regeneration processes, distribution, and safety duplication. The platform supported the management of large datasets and facilitated data exchange through standardized spreadsheet-based tools. Institutional customization included the development of modules for mobile data capture and workflow-specific operational tracking.

The collection is currently transitioning to the GRIN-Global Community Edition (GGCE). This upgraded platform provides enhanced inventory control functionalities, enabling real-time tracking of germplasm movements across conservation workflows and precise identification of the physical location and status of each conservation unit.

In addition to supporting an internal documentation system for germplasm management, efforts have been undertaken to ensure public accessibility of accession-level information to facilitate germplasm selection and distribution. Collection data were initially made available through the institutional genebank website. Currently, accession passport data and associated characterization and evaluation information are publicly accessible through Genesys (www.genesys-pgr.org/c/cassava), a global portal for plant genetic resources.

## 9. The Core Collection as a Tool for More Effective Conservation, Evaluation and Use

### 9.1. Purposes and Procedures

By the second half of the 20th century, many genetic resources programs established or expanded ex situ collections, but breeders often gave low priority to broad-scale evaluations of less-adapted germplasm. The core collection concept addressed the interest in streamlining and prioritizing conservation and evaluation, especially in large collections. Originally conceived by Frankel [93], a core collection would represent, “with a minimum of repetitiveness, the genetic diversity of a crop species and its wild relatives.” These collections are normally 5–10% of the total.

The CIAT cassava core collection of 630 accessions was developed at a time (mid-1990s) when a reasonable level of agronomic evaluation existed for the collection, but only rudimentary molecular data were available [94]. Criteria for inclusion in the core were based on four major types of information. Within these types, accessions were randomly chosen for inclusion in the core, except where otherwise indicated (because some accessions were included in multiple criteria, the sum of percentages is greater than 100):

- Base number of landrace accessions. As a starting point, the accessions from each country that were known to be landraces were flagged as candidates for the core; accessions from most countries are highly represented by landraces. Based on somewhat subjective criteria, the number of landraces was adjusted by an estimated proportion of duplicates for each country.
- Adjustments based on diversity of origins. There are a few source countries that make up most of the collection (Colombia, Brazil, Venezuela, Peru, Paraguay). However, many countries with smaller representation in the bank should also be important sources of diversity. To give them representation, they were weighted more heavily in the core, i.e., inversely related to the country’s collection size. This part of the core collection consists of 440 accessions (70% of the total core).
- Diversity of morphological characteristics. A total of 100 accessions (16% of total) were chosen based on their inclusion in the in vitro pilot genebank, which had been selected during the early experimental phase of the in vitro bank development, based on multiple diversity criteria.
- Diversity of alpha-beta esterase banding patterns. At the time of the core definition, the only available molecular classification was with the alpha-beta esterase system. Fifty-one accessions (8% of the core) were selected based on diversity within this group.
- A priori (non-random) selection based on predetermined criteria. Selection was based on multiple criteria of agronomic diversity and geographic origin (e.g., studies from the Cassava Biotechnology Network; landrace varieties of widespread use; and selection as elite clones (landraces or hybrids) by CIAT or IITA). This group includes 121 accessions (19% of the core).

Table 8 shows the representation of each country in the core, and on which criteria. More details are available in [94].

### 9.2. Genebank Management Implications

Establishment of the core had a series of implications for genebank management at CIAT. Defining the core helped highlight some of the gaps in the collection. For several countries with likely moderate-to-high genetic diversity, there are few accessions, which created somewhat of a challenge for how these countries should be represented and balanced against others in the core. Examples are Bolivia, Dominican Republic and Puerto Rico. Some countries with probable high cassava diversity currently have no representation in the core, such as the Guianas.

The main conservation implication of the core collection to date has been the prioritization for cryopreservation. After development of cryo-techniques with good recovery results, CIAT proceeded to first apply those protocols to the core.

From the time of core establishment, several projects have prioritized it to gain insights into genetic diversity of the whole collection. Among the first of these was the evaluation of several root and leaf quality traits [95]. These traits are based on time-consuming lab analyses, so evaluation of the core was a logical starting point. Over subsequent years, the core has also been prioritized as an effective means to estimate total diversity for several pest and disease resistance evaluations.

Rare or geographically restricted alleles may not be represented in the core collection. We cannot necessarily assume that a trait does not exist in the whole collection if it does not appear in the core. As molecular tools become more precise and more cost-effective, there will be a greater possibility to extend searches beyond the core to the entire collection for rare alleles, without the initial high costs of field evaluation.

Elias et al. [96], based on molecular markers, concluded that the CIAT core collection poorly represents the Amazon and Orinoco basins and the diversity of indigenous communities. This premise could certainly be a concern, and merits further study. However, only 59 accessions from a total of about 907 CIAT accessions from indigenous communities in the Amazon and Orinoco basins of Colombia and Peru were included and analyzed for this study. That representation is perhaps too small to conclude broadly that the core does not adequately represent the diversity of these regions.

### 9.3. Sharing the CIAT Core Collection Internationally

A report of the *Manihot* Genetic Resources Network [35] suggested sending the CIAT core collection to Brazil. However, Brazil was more focused on creating a core collection from their own substantial collection and therefore chose not to receive the one from CIAT. Longer-term plans call for Africa to also receive this sub-collection, when the introduction of vegetative material is more readily accepted.

In 2001, CIAT and the Thailand Department of Agriculture (DOA) jointly agreed to introduce the core collection to the Rayong Research Center of the DOA. As an indication of the complexity of international shipments of clonal cassava material, even for an advanced technology country like Thailand, this introduction of 628 accessions took five years to complete—from 2002 to 2007—and was done in 14 in vitro batches. Nearly all the clones were successfully passed through quarantine and established in the field. The DOA then characterized these accessions and evaluated them for an array of quality traits. The characterization data are available on Cassavabase.org.

Part of the core collection was also introduced to Vietnam and to Laos, with the main goal of evaluating these materials for possible resistance to cassava mosaic disease (after the reported presence of the causal virus in SE Asia in 2015) and to cassava witches’ broom disease. Three shipments from CIAT, totaling 232 accessions from the core, were sent in 2020 and 2021. Mortality in the first two shipments was high, and final overall survival was only 131 accessions, or 21% of the core. In this sense, the group of materials being evaluated in Vietnam could be considered *a* mini core, but not one based on specific criteria. At the time of writing this manuscript, evaluation in Vietnam is underway, especially for CMD resistance. The introduction to Laos was less successful and next steps are being planned (J. Newby and Thuy C., personal communication).

In 2024, 185 accessions from the core collection were distributed to the Chinese Academy of Tropical Agricultural Sciences (CATAS) for the purpose of evaluation and possible use in breeding (along with 256 landrace accessions and hybrids not in the core).

### 9.4. The Future of Cassava Core Collections

CIAT understood from the outset that the criteria for defining the original core collection in 1994 were far from ideal, in that they were based on somewhat subjective and low-precision assessments of genetic diversity. Nonetheless, there is a certain historical momentum that has occurred with this set of clones. More than 30 years after the definition of a core collection that was based on rather rudimentary criteria, the varietal components remain now just as they originally were selected. As more discriminating data become available, there are increasing arguments to redefine the core. For example, Santos et al. [97], based on the Brazilian cassava collection, provide suggestions on how to integrate morphological and agronomic traits, along with molecular markers, to best define a core.

The ideal core collection would be a global one, including material from multiple collections around the world, duplicated and accessible in several institutions. Currently, the exchange of vegetative material between Africa and the Americas significantly limits this option, but it can be an eventual goal.

## 10. Adding Value to Cassava Genetic Resources Through Their Use in Variety Development

Greater details about exploiting value of the cassava germplasm collection are covered in Part II of this series and are presented here as an introduction only.

### 10.1. General Principles

Over its 50-year history, use of the CIAT cassava collection has largely focused on the original purpose described by CIAT’s founders in 1969 [32]: “as a source of genes to improve cassava traits of grower and consumer interest, through selection and breeding.” This has involved a continual learning and innovation process for genebank managers, breeders and others who aim to understand and apply appropriate criteria for variety acceptance and adoption. Hershey [98] provides a broad overview of the historical process and progress in cassava breeding, including a review of the use of genetic resources.

The value of a crop germplasm collection is generally measured through its *use* as a global public good—past, present and future—not as a static museum display. From this perspective, the CIAT cassava collection has convincingly proven its value, and promises a high likelihood of continuing to do so. Understanding the proper balance of emphasis to give to different aims for germplasm use is an ongoing evolutionary process for research planners.

Cassava germplasm collection by CIAT, beginning in the late 1960s, coincided generally with the establishment of several national breeding programs around the world, and the renewal of some existing ones. For most of them, there were no bred varieties available. Breeders had no choice but to build from a base of landraces. Fifty years later, this situation and decision process about the use of landraces from genebanks offer many alternative options. There are six main steps in the pathway of extracting value from existing genebanks to create impact through new varieties:

(1) Gaining knowledge about traits of value, especially from end-users, which contribute to breeding program goals.
(2) Distributing accessions with desired traits, or their progeny, to partners.
(3) Incorporating target traits from the collection into candidate varieties, usually over generations of recurrent selection, but more recently through gene editing options.
(4) Full testing over years and locations representative of client farmers, and with consumers.
(5) Varietal release, multiplication, distribution—according to country norms and protocols.
(6) Farmer adoption at scale, driven ultimately by consumer demand.

### 10.2. Local and International Access and Use System for the Genebank Accessions

Access to genebank accessions for users outside of Colombia involves a complex system of infrastructure, technical capacity, institutional support, and an appropriate set of goals for their use. Most of the cassava genebank accessions fall under the Multilateral System of the International Treaty on Plant Genetic Resources for Food and Agriculture (ITPGRFA) [99]. Access to the germplasm is regulated by the Standard Material Transfer Agreement (SMTA) under Article 12.4 of the Treaty for the purpose of research, breeding and training for food and agriculture. Legally, accessions that are part of the Multilateral System are fully available for sharing and free use for all signatories of the Treaty. In practical terms, not all accessions are always available, due to conservation considerations such as ongoing pathogen cleaning, renovation or other temporary operational issues. The Genesys database is continuously updated to reflect the availability of accessions for shipment.

### 10.3. Distribution of Landraces and Their Hybrid Derivatives to Partners

Sharing alleles of value in the germplasm collection with partners and other interested entities is one of the genebank’s core activities. This happens in several ways, sometimes as in vitro cultures of genebank accessions, but most commonly in indirect form, as a hybrid seed that includes landrace varieties in the pedigrees. The genetic components of the collection have been shared through thousands of in vitro cultures and hundreds of thousands of seeds over the past 50 years.

**Figure 7:**
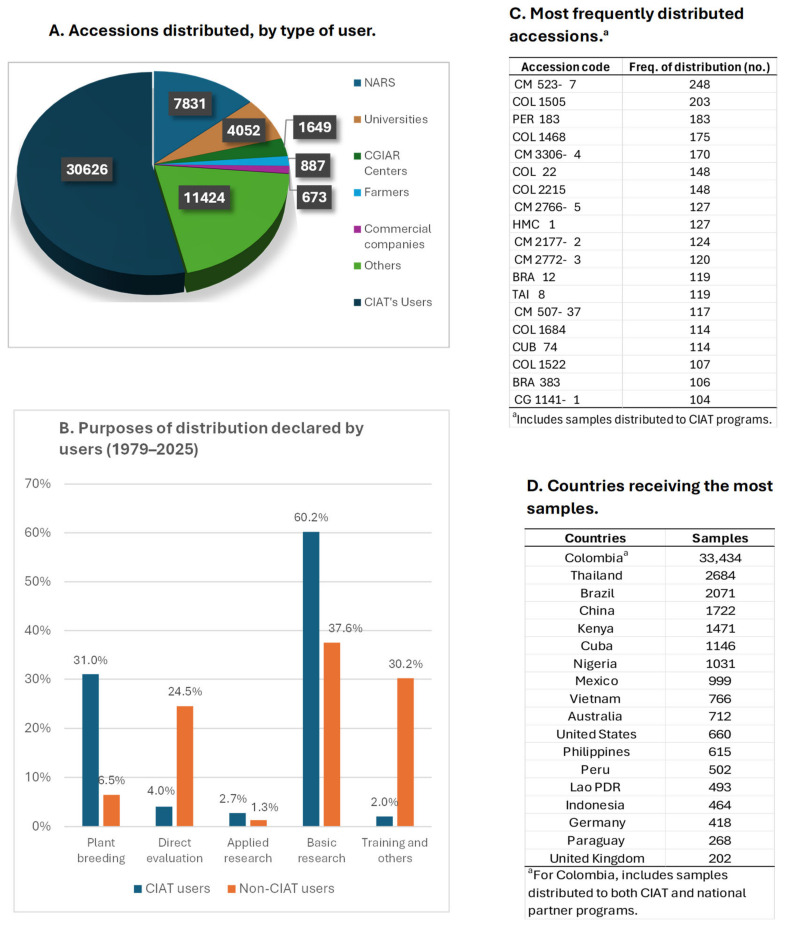
Distribution of cassava germplasm by CIAT Genetic Resources Program from 1979 to 2025.

Figure 7 summarizes the history of in vitro shipments from 1979 to 2025—a total of 30,626 in vitro samples to CIAT users and 26,516 to non-CIAT users. Outside of CIAT, the main users were partners in National Agricultural Research Systems, at 7831 samples. Around the year 2000, distribution accelerated when the collection was certified against diseases of quarantine importance.

The main purposes of external germplasm requests were direct evaluation of accessions for their value per se or for breeding, basic research (e.g., cryopreservation, embryogenesis, genomics and gene editing), and training. Some of the largest shipments were for the purpose of introducing the core collection to Thailand and Lao PDR.

Two accessions were distributed more than 200 times: the landrace variety COL 1505 and the CIAT hybrid, CM 523-7. Another 17 accessions were distributed more than 100 times. CIAT projects received just over half of the distributed accessions, while external institutions and partners received 46%. Four shipments, to Peru, Paraguay, Cuba and Ecuador, were carried out to restore lost germplasms to countries of origin.

## 11. Conclusions

### 11.1. Collection and Conservation

Further collection seems fully warranted, and the means of establishing logical priorities are now at hand. Some of the gaps are already known based upon important areas of cassava diversity that have not been adequately covered (or not covered at all) by past collections, e.g., Guyana, French Guiana, Suriname and Bolivia. The already-completed genotyping of the collection will allow a more sophisticated gap analysis as a reasonable second step to determine priorities. CIAT and IITA will together define patterns of diversity according to geographic origins, and from that, extrapolate areas needing further collection. Coordinated collection in Asia and Africa, especially of older landraces, will make important contributions to global knowledge and to sources of traits for breeding. This will be a long and complex process (perhaps in 15–20 years?), but with potentially high-value end products.

Consolidating all landraces on a global basis into a unified collection, with security backup, can act both in support of conservation in perpetuity of cassava genetic resources, and their effective use toward improved varieties.

The principal bases for current cassava conservation are via slow-growth in vitro, including security backups, and a parallel bonsai duplicate. Nonetheless, as a system looking toward conservation in perpetuity, added security and cost savings are warranted. The fine-tuning of cryopreservation seems to be at hand, with the likelihood of being able to move the entire collection into a more secure, lower cost conservation system.

Towill and Walters [100] discussed the possibility of a *doomsday* cryovault along the lines of the one for orthodox seeds at Svalbard, and in 2017 an international feasibility study of independent experts proposed such a vault for clonal crops [67]. In 2025, CGIAR introduced the Genebanks Accelerator as part of its new research portfolio, to modernize and expand the role of its global genebanks. It builds on decades of conservation work and positions genebanks not just as repositories of crop diversity, but as engines of innovation, resilience, and equitable access to genetic resources. In the case of clonal crops, inter-center and partner collaboration in cryopreservation is a key initiative. CIP has recently inaugurated a cryovault aimed at consolidating cryopreserved backups for several vegetatively propagated crops, including cassava.

The Crop Trust has made commitments to several crops, including beans at CIAT, for funding conservation in perpetuity. A similar funding commitment for cassava will be a strategic investment in one of the world’s most important crops.

A backup seedbank (botanical seeds), both at CIAT and in Svalbard, could be contemplated. However, without a field-based collection currently at CIAT, generating those seeds (ideally from self-pollination of each accession) would involve considerable cost and complexity. If seed production is to be accomplished, it would most likely be as an adjunct activity to some other initiative involving a field planting of the cassava collection.

Conservation of the wild *Manihot* species—in vitro (slow growth or cryo), as a bonsai planting, in the field and as seeds—is complex and difficult. Without their inclusion in Annex 1 of the International Treaty, their conservation remains at risk, and new collection missions are more difficult. In situ conservation, while not generally a priority within the CGIAR system, will also be extremely important to protect the wild species in their broadly at-risk native environments.

### 11.2. Characterization and Evaluation

For characterization, focus now shifts to full analysis of genotyping results. An early application is projected to be for duplicate identification [10]. To the extent that this involves confirmation with side-by-side comparisons in the field, the full process is likely to involve several years. It is still too early to precisely determine a duplication level in the collection, but this will become progressively clearer.

The genotyping data can be used to verify the validity of the selection of accessions for the core collection. There will need to be some balance of weight given to any newly arising information about diversity that is over- or under-represented in the current core collection, versus the fact that the current core already has a long history of associated evaluations.

Data recovery and updating from some early evaluations (early 1970s) is an ongoing, slow process. Within a year or two, and after all reasonable efforts of recovery, CIAT should be able to close the books on this process, and assume that archival data not already discovered are unlikely to become available in the future.

Conventional field evaluation for standard agronomic and quality traits of the current genebank accessions will have limited continuing utility for breeding programs. If the IITA and CIAT collections are eventually merged, then field evaluation could involve a major investment of resources for cross-continental trait discovery.

### 11.3. Exchange and Use

The exchange and use of genebank accessions rely fundamentally on the assurance of pathogen-free samples. Viruses are still the key challenge. Diagnostics and cleaning are becoming progressively more precise, allowing quarantine agencies to open avenues for further exchange. On the other hand, greater precision in viral identification, or even viral RNA incorporated into plant genomes, has been a double-edged sword. On the one hand it supports quarantine agencies in their goal of reducing pathogen threats. On the other hand, the increasing ability to detect viruses or segments of viral RNA that do not present a quarantine threat can very much slow down and complicate the legitimate exchange of germplasm.

Since about 40 years ago, the germplasm collection has been used mainly as a second-tier resource, after the evaluation of breeding lines reveals a lack of sufficient genetic diversity for target traits. If the breeder can access traits within improved lines, it is generally a more efficient pathway than the process of pre-breeding to bring those same traits from landrace varieties. This is even more so the case for the wild species: recovery of traits from wild species into advanced breeding lines is slow and difficult, even with advanced breeding tools. However, there are some areas where full evaluation of the collection will continue to be justified, especially targeting newly arising pest or disease issues, fine-tuned quality traits, and biochemical traits such as for pharmaceuticals or biologicals.

Some breeders envision a day when casava varieties will be developed through an inbred–hybrid breeding system, for example, [101]. This would allow taking full advantage of heterosis (maximized by crossing two fully homozygous lines). Additionally, it will allow much greater control by the breeder over trait integration into varieties that are already high performing in most traits. The development of an inbred–hybrid breeding system will need support from collection diversity analysis, and identification of heterotic groups.

### 11.4. Investment in the Future

The CIAT cassava collection has historically provided a very high return on investment in breeding. When the collection was established, very little breeding had been done on the crop. There were few bred varieties and an insignificant area planted to them, relative to the total production area. The genetic gap between bred varieties and landraces was very small.

However, the recommendation of landraces directly as new varieties was a short-lived process. Their use mainly as parents in breeding was already extensive within just a few years of the genebank’s establishment. Even in breeding, genetic gains attributable to continuing direct use of accessions as parents greatly declined from the 1990s onward. There were, however, key traits such as amylose-free (waxy) starch, and resistance to cassava mosaic disease and cassava brown streak disease that emerged from new evaluations in the 2000s. The collection has not been planted in the field for the past 20 years, and it has not undergone comprehensive field evaluation of any traits since that time. There have only been ongoing evaluations from small subsets of the collection, especially focusing on the core collection.

The collection has held relatively steady numbers over about 25 years—in the 5000-accession range for landrace varieties. The large burst in growth of Latin American materials during the initiation of collection in 1969/70 is not going to be repeated with new collections. However, it is reasonable to expect that some valuable new diversity can be identified and incorporated into the bank from the Americas. Much more significant gene bank growth would occur if the addition of African and Asian landraces was to become a reality. The process of introduction for both Africa and Asia would involve a multitude of science- and policy-based decisions and resources allocated, especially including quarantine protocols. This exchange among continents, preferably in both directions between CIAT and IITA, would open significant avenues of opportunity for collaboration in the secure conservation and effective use of a truly global collection of cassava genetic diversity.

CIAT’s Future Seeds genebank facility is giving new life to the conservation, evaluation and use of beans, forages and cassava. The new complex, inaugurated in 2022, and fully functional since 2024, integrates leading-edge conservation facilities with center-wide upstream and downstream research, along with partner training and collaboration, which ultimately provide sustainable benefits to farmers and consumers.

For cassava, Future Seeds offers greater security of conservation, emerging better knowledge of the landrace varieties that gave rise to the modern versions of this critical staple crop, and more possibilities to expand acquisition for a truly global collection. The genebank is aiming toward a fully cryopreserved cassava collection as a landmark step toward high-level long-term security. Integrating genomics information with field and lab experience will provide a further boost to fully exploiting the genebank to meet the demands arising from climate change and challenges to natural resource conservation, urbanization, mechanization and evolving consumer needs.

## Figures and Tables

**Table 1:** Collections according to altitude ranges (m above sea level).

Altitude Range (masl)	No. of Accessions in Range	Countries/Regions of Origin
>=2000	11	Colombia: Antioquia, Nariño, Cundinamarca, Magdalena
1600–1999	151	Colombia: 135; Ecuador: 4; Peru: 12
120–1599	270	Colombia: 255; Ecuador: 6; Guatemala: 3; Mexico: 3; Venezuela: 3
800–1199	275	Brazil: 28; Colombia: 204; Ecuador: 14; Guatemala: 6; Mexico: 1; Peru: 16; Venezuela: 6
0–799	1784	(Most regions)
Altitude not registered	2614	

Data from www.genesys-pgr.org/c/cassava (accessed on 5 March 2025).

**Table 2:** Number of accessions in CIAT collection by biological status, according to decade of introduction.

Decade of Introduction	Landraces	Bred Lines	Wild Species	Total
1969–1978	1722	3	0	1725
1979–1988	2242	100	0	2342
1989–1998	576	123	504	1203
1999–2008	83	34	0	117
2009–2018	165	3	0	168
2019–2023	0	0	0	0
Unclassified	306	402	365	1073
Total	5094	665	869	6628

Source: www.genesys-pgr.org/c/cassava (accessed on 14 November 2023).

**Table 3:** Comparison of landrace varietal density across countries, represented in the CIAT cassava collection.

Country ^a^	Collection Sites per Country	Total Varieties in Study ^b^	Mean Variety Density per Collection Site ^c^	Max. Variety Density per Collection Site ^c^	Area of Production 1980 (1000 ha) ^d^	Hectares per Landrace Variety	Landraces per 1000 ha
Argentina	43	61	1.4	4	18	287	3.4
Colombia	639	1606	2.5	34	165	103	9.7
Ecuador	60	109	1.8	8	20	183	15.2
Guatemala	64	88	1.4	5	4	40	2.2
Mexico	29	37	1.3	2	1	26	37.0
Panama	9	10	1.1	2	1	104	10.0
Paraguay	89	169	1.9	12	171	1010	1.0
Peru	60	167	2.8	9	103	619	1.6
Venezuela	24	71	3.1	8	39	544	1.8
Total or mean	1017	2318	2.3		521	225	4.4

^a^ The largest producer in the region, Brazil, is not included because collection site coordinates are not available for many of the landrace accessions. ^b^ Includes only landrace varieties with registered collection site coordinates. ^c^ A collection site is typically a field or a few fields from an individual farm managed by a household, but may be a few adjoining farms in a village. Individual collectors varied somewhat in their collection procedures. ^d^ Source: FAOSTAT. The year 1980 was chosen as a relative midpoint in the collection and acquisition of accessions of landrace varieties.

**Table 4:** Examples of high on-farm cassava diversity based on number of collected landrace varieties in three Departments of Colombia.

Department	Site/Farm Name ^a^	Range of CIAT Codes	No. Collected
Amazonas	El Encanto (km 1)	Col 2166–2191	25
	El Ventura km 6	Col 2152–2164 + Col 2202 + Col 2211	16
	Vereda El km 6	Col 2068–2077 + Col 2203	11
Guainía	Bellavista	Col 2356–2371	16
	El Sejal	Col 2266–2281	16
	Sarrapio	Col 2384–2419	36
	Yuri Caño Boron	Col 2302–2333	32
Putumayo	Buena Vista 100	Col 2579–2593	15
	La Samaritana Kms: 10	Col 2594–2607	14
	San Marcelino Kms: 10	Col 2567–2578	12

Source: www.genesys-pgr.org/c/cassava (accessed on 17 March 2025). ^a^ These data must be interpreted with some caution. It is not always clear from the collectors’ notes whether a collection site is a single farm or multiple farms combined. Additionally, land tenure arrangements vary considerably, from sole to community ownership, such that it may be difficult to distinguish between a farm and community or village, especially in indigenous communities. The accessions analyzed for the table do not include institutional acquisitions.

**Table 5:** *Manihot* wild species and subspecies held in vitro at CIAT.

Species	Principal Country of Origin	Accessions In Vitro
*AES (aescolifolia)*	Mexico	4
*ALT (alutacea)*	Brazil	4
*ANM (anomala)*	Brazil	0
*BLO (brachyloba)*	Colombia	0
*CAE (caerulescens)*	Brazil	17
*CEC (cecrophila)*	Unknown	6
*CHL (cholorsticta)*	Mexico	2
*CTH (carthaginensis)*	Colombia	26
*DCH (dichotoma)*	Brazil	0
*EPR (epruinosa)*	Brazil	2
*FMT (filamentosa)*	Brazil	3
*FLA (esculenta* subsp. *flabellifolia)*	Brazil	170
*FRU (fruticulosa)*	Unknown	1
*GLA (glaziovii)*	Brazil	1
*GUT (guaranitica)*	Paraguay	0
*HAS (hastatiloba)*	Unknow	0
*IRW (irwinii)*	Unknown	1
*JAC (jacobinensis)*	Brazil	6
*JNP (janiphoides)*	Brazil	2
*LON (longepetiolata)*	Unknown	6
*ORB (orbicularis)*	Unknown	8
*PEL (peltata)*	Unknown	0
*PIL (pilosa)*	Unknown	1
*PNT (pentaphylla)*	Brazil	1
*PER (esculenta* subs. *peruviana)*	Brazil	87
*PSE (pseudoglaziovii)*	Brazil	1
*PUR (purpureo-costata)*	Unknown	0
*QPT (quinquepartita*	Brazil	0
*RUB (rubricaulis)*	Unknown	0
*SPR (sparsifolia)*	Brazil	2
*TPH (triphylla)*	Unknown	9
*TST (tristis)*	Brazil	16
*VIO (violacea)*	Brazil	1
	Total:	377

Source: www.genesys-pgr.org/c/cassava (accessed on 10 March 2026).

**Table 6:** Sources of the CIAT cassava collection, with estimated original numbers collected or acquired, current accessions in vitro, and causes of major losses, where reported.

Origin	Prefix of Accession Code	Original Total ^a^	Current In Vitro Total ^b^	Est. No. of Landraces in Current Total ^c^	Historical Causes of Quarantine and Field Losses ^d^
Argentina	ARG	129	115	108	
Bolivia	BOL	7	7	7	
Brazil	BRA	1453	1294	1167	
China	CHN	2	2	2	
Colombia	COL	2826	1932	1902	CBB ^e^, salt spots in field
Costa Rica	CR	189	182	182	
Cuba	CUB	77	75	73	
Dominican Rep.	DOM	5	4	4	
Ecuador	ECU	205	112	112	Undefined quarantine issues
Fiji	FJI	6	6	6	
Guatemala	GUA	92	81	81	
Indonesia	IND	254	247	225	
Malaysia	MAL	80	71	62	
Mexico	MEX	111	100	96	
Nigeria (IITA)	NGA	19	18	0	
Panama	PAN	141	42	42	CBB, salt spots in field
Paraguay	PAR	220	203	201	
Peru	PER	616	397	397	CCMD ^f^ & CBB in quarantine
Philippines	PHI	6	6	4	
Puerto Rico	PTR	102	15	15	CBB, salt spots in field
Salvador	SLV	8	8	8	
Thailand	TAI	31	30	4	
USA	USA	9	8	8	
Venezuela	VEN	350	231	231	
Vietnam	VNM	9	9	9	
CIAT hybrids	AM, CG, CM, CT, SG, SM	408	379	0	
Total		7355	5574	4946	

^a^ Estimated based on highest accession number within a country of origin, including both landraces and partner hybrids. ^b^ Based on www.genesys-pgr.org/c/cassava (accessed on 15 August 2025). ^c^ Other accessions are mostly CIAT or partner hybrids. ^d^ Discarded in quarantine or lost from the field collection since origin of collection in 1969. Postulated as most likely scenarios from CIAT Cassava Program Reports over multiple years. Later losses in vitro are typically less associated with country of origin. ^e^ Cassava bacterial blight. ^f^ Cassava common mosaic disease.

**Table 7:** Redundancy groups identified by two molecular marker systems and categorized by region and country of origin.

	SNP and SilicoDArT Group Codes, and the Number of Landrace Accessions That Appear Simultaneously in Both Groups ^a^												
SNP:	127	703	739	752	1182	2313	2443	2713	2729	2889	3094	4535	Country Total
SilicoDArT:	3139	517	1273	3217	4747	2459	4531	3924	3639	4480	3461	4313	
**South America**													
Argentina			15			1							16
Brazil	8	2	9		1	10		1		1	1	3	36
Colombia	2		2	2	32	10	39	2	19	1	35	81	225
Ecuador	1							1		18	1	1	22
Paraguay			2			1							3
Peru	6			1								1	8
Venezuela	1	1		7		4		3		3	19		38
**Subtotal:**	18	3	28	10	33	26	39	7	19	23	56	86	348
**Meso-America/Carib.**													
Costa Rica	3	10	1	9		2		9	1				35
Cuba	2			1									3
Guatemala	1	24		2									27
Mexico				14							1		15
Panama				3				3		3	2		11
Puerto Rico				2									2
Salvador	3	3											6
**Subtotal:**	9	37	1	31	0	2	0	12	1	3	3	0	99
**Asia/South Pacific**													
Fiji	1												1
Indonesia	14										1		15
Malaysia		1											1
Thailand	1												1
**Subtotal:**	16	1	0	0	0	0	0	0	0	0	1	0	18
**Total accessions:**	43	41	29	41	33	28	39	19	20	26	60	86	465
**Total countries:**	12	6	6	9	2	7	1	6	3	5	7	4	18

Source: Adapted from [10]. Supplementary data. ^a^ While the two marker systems strongly coincided in their grouping of accessions, small differences existed. Numbers shown here include only accessions appearing together across both marker systems.

**Table 8:** Basis for accessions selected for the CIAT cassava core collection. (Some accessions meet multiple criteria.)

Geographic Origin or Group	Base No. of Accessions at Time of Core Formation	Number of Accessions Selected Based on Various Criteria				
		Origins	Morphological Diversity	Diversity of Esterase Isozymes	A priori Selection	Final Number in core from Each Group ^a^
Argentina	16	2	4	0	3	8
Bolivia	3	1	2	0	3	3
Brazil	1637	110	13	15	20	101
China	2	1	0	0	2	2
Colombia	1907	111	15	13	14	142
Costa Rica	147	9	7	5	4	23
Cuba	74	10	5	1	2	18
Dominican Rep.	5	2	2	0	4	9
Ecuador	117	25	6	0	4	32
Fiji	6	1	0	0	2	2
Guatemala	91	8	6	0	2	15
Indonesia	51	1	0	2	5	7
Malaysia	68	8	0	1	6	15
Mexico	100	14	6	0	2	20
Panama	42	6	2	0	2	9
Paraguay	192	25	8	3	7	40
Peru	405	63	10	3	2	76
Philippines	6	1	0	0	2	2
Puerto Rico	15	1	2	0	4	7
Thailand	8	0	0	0	4	4
USA	9	0	0	0	4	4
Venezuela	240	41	9	3	3	55
CIAT hybrids	317	0	3	5	27	33
IITA hybrids	19	0	0	0	3	3
**Total**	**5477**	**440**	**100**	**51**	**121**	**630**

^a^ Since original development of the core collection, one accession has been lost from the in vitro collection.

## Data Availability

Genebank collection, conservation and characterization data are available primarily at https://www.genesys-pgr.org/wiews/COL003.

## Supplementary Materials

The following supporting information can be downloaded at: https://www.mdpi.com/article/10.3390/plants15131981/s1, Figure S1: Revised collection form for cassava. Table S1: Selected milestones related to establishment, management and use of the CIAT cassava germplasm collection. Table S2: Origins, dates, collectors and institutions for principal collections and acquisitions contributing to the CIAT cassava genebank over five decades. Table S3: Utility of conservation, distribution and evaluation options for alternative conservation methods of cassava and its wild relatives. Table S4: Composition of culture media used for in vitro conservation and. Table S5: *Manihot* species held as herbarium specimens at CIAT. Table S6: Cassava morphological descriptors as defined by Fukuda et al. (2010) [6]. Table S7: Putative simply inherited characters in cassava and their gene symbols. Table S8: Techniques and tools for molecular diversity analysis applied to the CIAT cassava collection.
