# Supplementary material for: Adding Value to Cassava Genetic Resources Conserved at CIAT—Part I: A Review of Fifty Years of Collection, Conservation, Characterization and Distribution
Source: Plants (Basel). 2026 Jun 26;15(13):1981. doi: 10.3390/plants15131981 (PMC13363913; doi:10.3390/plants15131981)
Supplement: Supplementary file 1 [file plants-15-01981-s001.zip › Supplementary Figure S1.pdf]

Supplementary Figure S1. Revised collection form for cassava.

MANIHOT COLLECTION FORM (part 1 of 2)

GENUS: _______________ **SPECIES**: ________________ **SUBSPECIES**:_________________

COLLECTORS' INITIALS: ____________________ **COLLECTION NUMBER**: ___________

INSTITUTION RESPONSIBLE: ____

DATE OF COLLECTION (Day/Month/Year): ____/____/____

COUNTRY OF COLLECTION: _________________ **PROVINCE/STATE**: _______________

LOCALITY:

Nearest town/village: ____

Distance (km): __________________ Direction from town: ________________________

LATITUDE: Degrees: ______ Minutes: ______ N S

LONGITUDE: Degrees: ______ Minutes: ______ E W

ALTITUDE: (m) ________

COLLECTION SOURCE (circled):

Wild	1	Village market	5
Farmer's field	2	Commercial market	6
Store	3	Institution	7
Backyard	4	Other: ______________	8

SAMPLE STATUS (circled):

Wild	1	Primitive variety/landrace	4
Weedy	2	Improved variety (breed)	5
Breeder's line	3	Other: ______________	6

LOCAL NAME: ____

NUMBER OF PLANTS SAMPLED: ______________ **PHOTOGRAPH** (circled): Yes No

TYPE OF SAMPLE: (circled):

Vegetative 1 Seed 2 Both 3

HERBARIUM SPECIMEN (circled): Yes No

QUANTITY OF MATERIAL (number of seeds, stem pieces, tubes *in vitro*): _____________

PRIMARY MORPHOLOGICAL DESCRIPTORS (cultivated cassava only) (circle number):

Colour of apical leaves: 3=light green; 5=dark green; 7=purplish green; 9=purple

Leaf lobe form: 1=linear; 2=elliptic; 3=lanceolate

Petiole colour: 1=yellowish green; 2=green; 3=green with slight red; 5=green with red; 7=red; 9=purple

Apical pubescence: 0=absent; 3=little; 5=moderate; 7=high

Stem epidermis colour (internal surface): 1=silver green; 2=light brown/orange; 3=dark brown

Stem periderm colour: 1=light green; 2=dark green; 3=yellow

Root surface colour: 1=white or cream; 2=yellow; 3=light brown; 4=dark brown

Root flesh colour: 1=white; 2=cream; 3=yellow; 4=pink

Flowering: 0=absent; 1=present

Storage root peduncle: 1=sessile (absent); 2=short (<5cm); 3=intermediate/long (>5cm)

Root cortex colour: 1=white or cream; 2=yellow; 3=pink; 4=purplish

MANIHOT COLLECTION FORM (part 2 of 2)

GROWTH HABIT (circled):
Tree 1 Bush 2 Vine 3 Other ________________________________
PART OF PLANT UTILIZED (circled): Roots 1 Foliage 2
PRINCIPAL USE (circled):

Human consumption - fresh	1	Animal consumption - dry or processed	4
Human consumption - dry or processed	2	Starch extraction	5
Animal consumption - fresh	3	Other ___________________________	6

SPECIAL QUALITIES (according to farmer) (circled):

Yield	1	Disease resistance	5
Starch content	2	Pest resistance	6
Eating quality	3	Edaphic adaptation	7
Root storability	4	Other ________________	8

NOTABLE DEFECTS (according to farmer): ____

DISEASES AND PESTS PRESENT AND SEVERITY:
(Severity: 1=little damage; 2=moderate damage; 3=severe damage)

Diseases/Pests	Severity	Diseases/Pests	Severity
___________________________	______	____________________________	______
___________________________	______	____________________________	______
___________________________	______	____________________________	______

WILD SPECIES AND ASSOCIATED CROPS:
______________________________________ ______________________________________
______________________________________ ______________________________________

TOPOGRAPHY (circled):

Marshy	1	Rolling hills	5
Flood plain	2	Steep hills	6
Riparian	3	Mountainous	7
Flat – not flood-prone	4	Other ________________	8

VEGETATION (circled):

Rainforest	1	Thorn woodland	6
Humid forest	2	Scrub desert	7
Semi-humid forest	3	Desert	8
Dry forest	4	Other ________________	9
Very dry forest	5		

SOIL TEXTURE (circled):

Sandy	1	Clayey	5
Sandy loam	2	Silt	6
Loam	3	Organic origin	7
Clay loam	4	Other ________________	9

DRAINAGE (circled):
Poor 1 Moderate 2 Good 3 Excessive 4

SLOPE (circled):

Flat or almost flat	($< 4^o$)	1
Moderate slope	(4-14°)	2
Steep slope	($>14^o$)	3

____

Source: Adapted from Gulick et al. (1983) [34]; IPGRI (1994) [35]
