# Supplementary material for: Adding Value to Cassava Genetic Resources Conserved at CIAT—Part I: A Review of Fifty Years of Collection, Conservation, Characterization and Distribution
Source: Plants (Basel). 2026 Jun 26;15(13):1981. doi: 10.3390/plants15131981 (PMC13363913; doi:10.3390/plants15131981)
Supplement: Supplementary file 1 [file plants-15-01981-s001.zip › Supplementary Table S1.pdf]

Supplementary Table S1. Selected milestones related to establishment, management and use of the CIAT cassava germplasm collection.[a]

1969-1970	Victor Manuel Patiño of the Valle Botanical Garden led several collection expeditions throughout South and Meso-America to initiate the first cassava genebank for CIAT.
1970-1974	A collection of about 2000 accessions was established in the field at CIAT-Palmira, with main representation from Colombia, Venezuela, Ecuador, Peru and Mexico. Accessions from Brazil, well-recognized as a major center of diversity, could not be introduced early on because of the risk of introducing coffee rust spores, as well as the widespread presence of cassava common mosaic disease.
	Challenges of cassava common mosaic and cassava bacterial blight caused significant losses during quarantine and in the field genebank, especially in the Peruvian accessions.
	Cassava staff expanded through the hiring of a Cassava Program leader/physiologist (James H Cock) in 1972, followed by a breeder in 1973 (Kazuo Kawano), and in quick succession thereafter Agronomist (Julio Cesar Toro), Utilization Specialist (Julian Buitrago), Entomologists (Aart van Schoonhoven and Tony Bellotti).
1973	First activity of the new breeder, was to evaluate the collection at the CIAT Palmira station, under conditions of good soils, moderate temperatures, isolation from other cassava plantings (in the middle of extensive cane fields).
	Initial collection evaluation, based largely on the history of improving major grains by increasing harvest index (proportion of biomass of commercial plant part to total biomass), along with root dry-matter content.
1974	Successfully selected 230 accessions as the first round of elite materials from the collection.
1975	Hybridization to recombine best traits from selected accessions. This became the basis for the first era (approximately a decade) of CIAT cassava breeding.
1974-1985	Extensive screening by Pathology and Entomology sections, with emphasis on greenhouse/screenhouse plantings, for potential resistance to major pests and diseases known at the time. (Later, this would evolve to mainly field evaluations.)
	Physiology group shows importance of daylength and temperature on cassava growth and development, and the effect of evolutionary origins on adaptation.
	An *ideal plant type* for productivity is defined. A given variety may vary its plant type depending on the testing environment, illustrating one factor in the importance of testing in the target production environment.
1978	Breeder hired with one of key goals to further understand and exploit collection accessions for breeding, and to support the filling of gaps in the collection.
	Genetic Resources Unit established at CIAT, which would gradually take over various responsibilities from Cassava Program for genebank management.
1978-1985	A series of studies led by Plant Pathology and Entomology sections over several years and environments confirms the strong GxE interaction for variety performance, and the distinctive region-specific pest and pathogen complexes.
	Emphasis on full collection evaluation across diverse agroecological sites (as opposed to previous strategy of initial elimination based on CIAT-Palmira performance).

Supplementary Table S1. Selected milestones related to establishment, management and use of the CIAT cassava germplasm collection.[a]

	Diversity of landrace parents in breeding program increased as evaluations in multiple environments progressed.
1979	William Roca, Physiologist, hired to establish the cassava *in vitro* collection and provide support to pathogen-free international germplasm movement; later, with broader responsibilities in genetic resources management.
1979-1982	*In vitro* techniques allow a new round of introductions, including from Brazil, which previously had very few representatives.
1983-1986	Biotechnology Research Unit formed, led by William Roca, with a key purpose of managing the *in vitro* cassava collection, including international distribution.
	First accessions introduced from Asia (Malaysia).
	First introductions from Africa (IITA, Nigeria) through intermediate quarantine in the Scottish Crop Research Institute to doubly verify freedom from cassava mosaic virus. Main purpose was for CIAT to have direct access to sources of resistance to cassava mosaic disease, and thereby to facilitate introduction of greater genetic diversity to Africa.
	Systematic protocol for *in vitro* conservation established; cryopreservation successfully tested.
1990	Accessions added progressively, with about 70% of the current collection by 1990.
1992-1995	Based on prior pilot work on wild *Manihot* species propagation (seed and *in vitro*), and with IBPGR funding, about 30 species introduced as either *in vitro* or seed material, mainly from Brazil. Difficult to maintain either in the field or *in vitro*. Limited but important evaluations and crossing with cassava.
1993	Core collection developed based on best available information on passport, characterization and evaluation data (and including basic information on isozymes, but prior to other more discriminating molecular tools).
1994	Formation of the International Network for Cassava Genetic Resources by IBPGR.
1997	A pioneering study on *Manihot* diversity based on molecular markers (AFLPs) paves the way for many other molecular studies to come (Roa et al. 1997).[4]
1998	Cassava descriptors list, used and refined over 20 years at CIAT and at CNPMF/Embrapa, published by Fukuda and Guevara (1998) [5]. Translated and updated in 2010 by Fukuda et al. (2010) [6].
2000-2002	The collection became increasingly difficult to manage as a field collection, mainly due to cassava frogskin disease build-up. Eliminated in 2002.
2002	A potted plant (*bonsai*) back-up system created first for disease indexing, and converted to a low-cost alternative to the field genebank. It serves as an interim source of clean propagative material between *in vitro* conservation and field experimental use.
2005	A back-up safety system developed in agreement with the International Potato Center (CIP) and the governments of Colombia and Peru to improve conservation security.
2006	First high-value single gene trait – amylose-free starch (*waxy*) – discovered in CIAT collection by teams led by Hernan Ceballos, Dominique Dufour, Teresa Sanchez and colleagues (Ceballos et al., 2007) [7].

Supplementary Table S1. Selected milestones related to establishment, management and use of the CIAT cassava germplasm collection.[a]

2002-2012	Based initially on collection accessions, the search for high levels of carotenoids in cassava, and a breeding strategy to further increase them, contributes to global food security through biofortification.
2010	The Crop Trust publishes *A Global Conservation Strategy for Cassava and Wild Manihot Species* as part of the Trust's extensive review of strategies for global crops (Hershey, 2010) [8]
2012	CGIAR Research Program (CRP) established for system-wide integration of the CG genebanks, to be managed by the Crop Trust.
2013	External review team recommends that steps should be taken for CIAT to process entire cassava collection for cryopreservation.
2014	Identification of a potential complex of viruses associated with cassava frogskin disease, adding new complexity to germplasm indexing and sanitation protocols.
2019	Landmark discovery by Stephan Winters and team, Leibniz Institute (Germany), for resistance to cassava brown streak disease, currently reported only in East Africa, in several landraces from the CIAT collection (Sheat et al., 2022) [9]
2019-2023	Various donors provide funding to genotype by sequencing and to analyze the full collection. Early priorities include identification of duplicates, and associating collection site with specific climate adaptation, especially as a strategy for developing climate-smart varieties (Carvajal et al., 2024) [10].
2022	CIAT inaugurates Future Seeds as a state of the art genetic resources management facility, including *in vitro* conservation (slow growth and cryo) of cassava as a vegetatively propagated species.
