# Supplementary material for: Adding Value to Cassava Genetic Resources Conserved at CIAT—Part I: A Review of Fifty Years of Collection, Conservation, Characterization and Distribution
Source: Plants (Basel). 2026 Jun 26;15(13):1981. doi: 10.3390/plants15131981 (PMC13363913; doi:10.3390/plants15131981)
Supplement: Supplementary file 1 [file plants-15-01981-s001.zip › Supplementary Table S2.pdf]

**Supplementary Table S2**. Origins, dates, collectors and institutions for principal collections and acquisitions contributing to the CIAT cassava genebank over five decades.[a]

| | Years of intro. to CIAT | Range in accession numbers (no. surviving in genebank as of 2023) | From prior institutional collection (I) or de novo collection expedition (E) | Collectors or contributing institutions |
|---|---|---|---|---|
| **Argentina** | | | | |
| | 1983 | 1-16 (16) | I | ? |
| | 1994-1995 | 17-107 (84) | E | Carlos Iglesias, Irland Luis Gnoato, Miguel Elechosa |
| | 2002 | 108-117 (10) | ? | ? |
| | 1994 | 118-129 (12) | I | Instituto Nacional de Tecnología Agropecuaria |
| **Bolivia** | | | | |
| | 1977 | 1-3 (3) | I | Estacion Expt. Agricola de Tulumayo, Peru |
| | 1995 | 4-7 (4) | I | Universidad Autónoma Gabriel Rene Moreno |
| **Brazil** | | | | |
| | 1977-1979 | 1-54 (43) | I | Unknown |
| | 1979 | 55-56 (11) | I | CNPMF |
| | 1977 | 28-37 (9) | I | Estacion Expt. Agricola de Tulumayo, Peru |
| | 1980 | 67-188 (121) | I | EMBRAPA/CNPMF |
| | 1981-1982 | 189-354 (166) | I | CNPMF |
| | 1982 | 355-358 (3) | I | EMBRAPA/Centro de Pesquisa Agroforestal da Amazonia Orienal |
| | 1982 | 359-451 (91) | I | Empresa Goiana de Pesquisa Agropecuaria |
| | 1982 | 452-522 (69) | I | Unidade de Experimentacao e Pesquisa de Âmbito Estadual do Acre |
| | 1982 | 535-607 (72) | I | Instituto de Pesquisa Agronomica de Estado do Rio Grande do Sul |
| | 1983 | 609-770 (104) | I | Empresa Estadual de Pesquisa Agropecuaria de Santa Catarina |

**Supplementary Table S2**. Origins, dates, collectors and institutions for principal collections and acquisitions contributing to the CIAT cassava genebank over five decades.[a]

| | Years of intro. to CIAT | Range in accession numbers (no. surviving in genebank as of 2023) | From prior institutional collection (I) or de novo collection expedition (E) | Collectors or contributing institutions |
|---|---|---|---|---|
| | 1983 | 773-806 (26) | I | EMBRAPA/Centro de Pesquisa Agropecuaria dos Cerrados/CNPMF |
| | 1983 | 859-865 (5) | I | Empresa Estadual de Pesquisa Agropecuaria de Santa Catarina |
| | 1980 | 842-850 (9) | I | ? |
| | 1988 | 875-892 (17) | I | EMBRAPA/CNPMF |
| | 1988 | 893-900 (8) | I | Empresa Estadual de Pesquisa Agropecuaria de Santa Catarina |
| | 1988 | 900-928 (28) | ? | Unknown |
| | 1991 | 932-1303 (340) | I | CNPMF |
| | 1992 | 169 | I | EMBRAPA/CNPRGB |
| | 1992-1994 | 1304-1452 (135) | I | EMBRAPA/CNPMF |
| **Colombia** | | | | |
| | 1969-1970 | 1-1821 (1171) | E | Victor Manuel Patiño, Pablo Emilio Daza |
| | 1971 | 1822-1830 (9) | E | Pablo Daza |
| | 1975 | 1886-1939 (48) | I | ICA |
| | 1978 | 2002-2019 (14) | E | Clair Hershey, J Carlos Lozano |
| | 1979 | 2024-2057 (33) | E | Octavio Vargas, Pedro Millan, Rafael Laberry |
| | 1979 | 2058-2067 (10) | E | Rafael Laberry, J Carlos Lozano, Jairo Castano |
| | 1979 | 2068-2214 (143) | E | Alvaro Amaya, Clair Hershey, Pablo Emilio Daza, Yoshiki Umemura |
| | 1980 | 2204-2207 (4) | E | Pedro Millan |
| | 1981 | 2217-2245 (21) | E | Margaret Jan Salick, Robert Zeigler |
| | 1986 | 2266-2419 (144) | E | Mario Mejia Gutierrez (U. Nacional de Colombia) |
| | 1986 | 2424-2519 (84) | E | Darna Dufour, P. Patmore, R.H. Wilshusen |

**Supplementary Table S2**. Origins, dates, collectors and institutions for principal collections and acquisitions contributing to the CIAT cassava genebank over five decades.[a]

| | Years of intro. to CIAT | Range in accession numbers (no. surviving in genebank as of 2023) | From prior institutional collection (I) or de novo collection expedition (E) | Collectors or contributing institutions |
|---|---|---|---|---|
| | 1987 | 2520-2621 (90) | E | Mario Mejia Gutierrez (U. Nacional de Colombia) |
| | 1987 | 2690-2716 (27) | E | Benjamin Pineda, Barry Nolt |
| | 1988 | 2717-2732 (16) | E | Benjamin Pineda |
| | 1991 | 2629-2688 (56) | I | ICA |
| | WITHOUT DATES | 1831-1885 (27) | I | |
| | WITHOUT DATES | 1940-2001 (51) | I | |
| **Costa Rica** | | | | |
| | 1975 | 1-16 (15) | I | ? |
| | 1983-1984 | 17-149 (132) | I | CATIE |
| | 2012 | 150-189 (40) | I | CATIE |
| **Cuba** | | | | |
| | 1979-1980 | 1-73 (73) | I | Centro de Mejoramiento de Semillas Agamicas (CEMSA) "Fructuoso Rodriguez" |
| **Dominican Rep.** | | | | |
| | 1975 | 1-5 (5) | I | ? |
| **Ecuador** | | | | |
| | 1970 | 1-123 (76) | E | Victor Manuel Patiño, Betancourt |
| | 1971 | 125-191 (37) | E | Victor Hugo Santillan, Victor Manuel Patiño |
| **Fiji** | | | | |
| | 1987 | 1-6 (6) | I | FAO |
| **Guatemala** | | | | |
| | 1984 | 1-92 (91) | E | Instituto de Ciencia y Tecnología Agropecuaria |

**Supplementary Table S2**. Origins, dates, collectors and institutions for principal collections and acquisitions contributing to the CIAT cassava genebank over five decades.[a]

| | Years of intro. to CIAT | Range in accession numbers (no. surviving in genebank as of 2023) | From prior institutional collection (I) or de novo collection expedition (E) | Collectors or contributing institutions |
|---|---|---|---|---|
| **Indonesia** | | | | |
| | 1986 | 1-48 (48) | I | Central Research Institute for Food Crops |
| | 2005 | 52-135 (84) | I | Central Research Institute for Food Crops |
| | 2009 | 136-254 (117) | I | Central Research Institute for Food Crops |
| **Malaysia** | | | | |
| | 1981 | 1-3 (3) | I | Malaysian Agricultural Research and Development Institute (MARDI) |
| | 1986 | 4-69 (64) | I | MARDI |
| | 2012 | 70-80 (11) | I | MARDI |
| **Mexico** | | | | |
| | 1970 | 1-66 (60) | E | Xolocotzi, Hernandez, Victor Manuel Patiño |
| | 1975 | 68-71 (4) | ? | ? |
| | 1987 | 72-111 (38) | I | Instituto Nacional de Investigaciones Forestales, Agrícolas y Pecuarias |
| **Nigeria** | | | | |
| | 1987-1988 | 1-19 (19) | I | IITA (via Scottish Crop Research Institute |
| **Panama** | | | | |
| | 1970 | 1-114 (22) | E | I. de Polanco, Victor Manuel Patiño |
| | 1985 | 119-138 (19) | I | Instituto de Investigaciones Agropecuarias de Panama |
| **Paraguay** | | | | |

**Supplementary Table S2**. Origins, dates, collectors and institutions for principal collections and acquisitions contributing to the CIAT cassava genebank over five decades.[a]

| | Years of intro. to CIAT | Range in accession numbers (no. surviving in genebank as of 2023) | From prior institutional collection (I) or de novo collection expedition (E) | Collectors or contributing institutions |
|---|---|---|---|---|
| | 1975 | 1-2 (2) | | |
| | 1983-1985 | 5-195 (189) | E | J. Gerardo Vieira, Luis Caceres, Alvaro Amayo Mario Sanabria, Tomas Mayeregger, Ministerio de Agricultura y Ganadería[b] |
| | 1992 | 198-220 (15) | I | Instituto Agronomico Nacional |
| **Peru** | | | | |
| | 1977 | 176-253 (75) | I | Estacion Expt. Agricola de Tulumayo, Peru |
| | About 1980(?) | 254-327 (68) | I | Centro Regional de Investigación Agraria CRIA |
| | 1983 | 329-454 (120) | I | Universidad Nacional Pedro Ruiz Gallo |
| | 1986-1987 | 455-606 (130) | E | Margaret Jan Salick, Sandra Knapp, Edward Carey |
| | 1987 | 608-615 (8) | E | Universidad Nacional Pedro Ruiz Gallo |
| **Puerto Rico** | | | | |
| | 1970 | 1-57 (14) | E | Victor Manuel Patiño |
| **El Salvador** | | | | |
| | 1996 | 1-8 (8) | I | Centro Nacional de Tecnología Agropecuaria y Forestal |
| **Thailand** | | | | |
| | 1986 | 2-8 (7) | I | Thailand Dept. of Agriculture |
| | 1990-1991 | 9-31 (23) | I | Thailand Dept. of Agriculture |
| **USA** | | | | |
| | 1986 | 1-9 (9) | I | United States Dept. of Agriculture |

**Supplementary Table S2**. Origins, dates, collectors and institutions for principal collections and acquisitions contributing to the CIAT cassava genebank over five decades.[a]

| | Years of intro. to CIAT | Range in accession numbers (no. surviving in genebank as of 2023) | From prior institutional collection (I) or de novo collection expedition (E) | Collectors or contributing institutions |
|---|---|---|---|---|
| **Venezuela** | | | | |
| | 1971 | 1-113 (85) | I | U. Central de Venezuela |
| | 1971 | 114-217 (83) | E | Jairo Quintero, Victor Manuel Patiño |
| | ? | 218-332 (73) | ? | ? |
| **Viet Nam** | | | | |
| | 1996 | 1-9 (9) | I | Vietnam Agricultural Science Institute |

Source: www.genesys-pgr.org/

[a]This list is not fully comprehensive. Some smaller collections, such as those that were not part of a planned and funded collecting expedition or institutional acquisition are not included here.

[b]Multiple expeditions with different collectors on each.
