# Supplementary material for: Adding Value to Cassava Genetic Resources Conserved at CIAT—Part I: A Review of Fifty Years of Collection, Conservation, Characterization and Distribution
Source: Plants (Basel). 2026 Jun 26;15(13):1981. doi: 10.3390/plants15131981 (PMC13363913; doi:10.3390/plants15131981)
Supplement: Supplementary file 1 [file plants-15-01981-s001.zip › Supplementary Table S3.pdf]

Supplementary Table S3. Utility of conservation, distribution and evaluation options for alternative conservation methods of cassava and its wild relatives.[a]

Activity	Field genebank	Bonsai	*In vitro*	Seed bank	Cryo bank	*In situ*
**CASSAVA**						
**Conservation**	Yes, with serious phytosanitary risks	Yes, with intermediate level phytosanitary risks	Yes, for medium term conservation	Yes, but for conservation of genes, not original landrace genoytpes	Yes, for long term conservation; for generating safety backup copies, would be done in large batches	Yes, but will require some management input and agreement among farmers and conservationists
**Routine distribution**	Within country only	Potentially safe, but with very stringent monitoring required; not currently approved	Safe, with full indexing	Safe, with standard seed treatments	Safe but complex; requires thawing and recovering of *in vitro* plants	No, except sharing as normally done among neighbors and communities
**Evaluation**	Immediate, or easily done with planting material taken to other sites	Partial; intermediate propagation phase required	Delayed due to slow recovery rate and hardening times	Typically not suitable unless large number (100s?) of seeds available	Yes, but much delayed for recovery and multiplication	Yes, but limited to site-specific evaluation; not suited to broad evaluation of the collection
**WILD SPECIES**						
**Conservation**	Very challenging for most species at Palmira station	Difficult to establish and maintain, poor rooting and growth	Yes, but modification to media required for some species	Yes	Little research done	Yes, but minimal efforts to date
**Distribution**	Risky (if field collection established)	No; distribution normally by seed	Only system currently used, but potentially difficult if recipients have inadequate facilities	Safe	Yes	No
**Evaluation**	Immediate	Limited	Delayed	Delayed	Much delayed	Limited potential due to scattered distribution

[a]Adapted from Mafla and Debouck, 2004 [53]
