# Supplementary material for: Adding Value to Cassava Genetic Resources Conserved at CIAT—Part I: A Review of Fifty Years of Collection, Conservation, Characterization and Distribution
Source: Plants (Basel). 2026 Jun 26;15(13):1981. doi: 10.3390/plants15131981 (PMC13363913; doi:10.3390/plants15131981)
Supplement: Supplementary file 1 [file plants-15-01981-s001.zip › Supplementary Table S4.pdf]

Supplementary Table S4. Composition of culture media used for *in vitro* conservation and micropropagation purposes of cassava collection at CIAT[a]

**Purpose**	**Medium**	**Composition**
Conservation/ Micropropagation	MS Murashige and Skoog (1962) [59]	Murashige and Skoog salts with vitamins, 3% sucrose, 0.7% BD Difco™ agar, pH 5.7-5.8
Conservation for cultivated accessions	NP (Mafla et al 2000) [60]	Murashige and Skoog salts, 2% sucrose, 1 mg/L thiamine hydrochloride, 100 mg/L myo-inositol, 0.02 mg/L 6-benzylaminopurine, 0.1 mg/L gibberellic acid, 0.01 mg/L naphthaleneacetic acid, 10 mg/L silver nitrate, 0.7% BD Difco™ agar, pH 5.7-5.8
Conservation for wild species accessions	12NP (Mafla et al 2000) [60]	Murashige and Skoog salts, 3% sucrose, 1 mg/L thiamine hydrochloride, 100 mg/L myo-inositol, 0.2 mg/L kinetin, 1 g/L activated charcoal, 10 mg/L SN, 0.7% BD Difco™ agar, pH 5.7-5.8)
Micropropagation	4E (Roca et al,1991) [61]	Murashige and Skoog salts, 2% sucrose, 1 mg/L thiamine hydrochloride, 100 mg/L myo-inositol, 0.04 mg/L 6-benzylaminopurine, 0.05 mg/ L gibberellic acid, 0.02 mg/L naphthaleneacetic acid, 0.7% BD Difco™ agar; pH 5.7-5.8
Micropropagation for wild species accessions	12A3 (Mafla et al 2000) [60]	Murashige and Shook salts, 3% sucrose, 1 mg/L thiamine HCl, 100 mg/L myo-inositol, 0.2 mg/L kinetin, 1 g/L activated charcoal, 0.7% BD Difco™ agar, pH 5.7-5.8
Rooting	17N (CIAT, 1982) [62]	1/3 Murashige and Shook salts, 2% sucrose, 0.01 mg/L GA_3, 0.01 mg/L NAA, 25 mg/L10-52-10 Plantafol® fertilizer, 2 g/L Phytagel™ agar; pH 5.7-5.8.

[a]The medium formulations are as follows, where MS = Murashige and Skoog basic salts:

NP: MS, 2% sucrose, 1 mg/L thiamine hydrochloride (HCl), 100 mg/L myo-inositol, 0.02 mg/L 6-benzylaminopurine (BAP), 0.1 mg/L gibberellic acid (GA_3), 0.01 mg/L naphthaleneacetic acid (NAA), 10 mg/L silver nitrate (SN), 0.7% BD Difco™ agar, pH 5.7-5.8), MS (MS with vitamins, 3% sucrose, 0.7% BD Difco™ agar, pH 5.7-5.8)

12NP: MS, 3% sucrose, 1 mg/L thiamine HCl, 100 mg/L myo-inositol, 0.2 mg/L kinetin, 1 g/L activated charcoal (AC), 10 mg/L SN, 0.7% BD Difco™ agar, pH 5.7-5.8

4E: MS, 2% sucrose, 1 mg/L thiamine HCl, 100 mg/L myo-inositol, 0.04 mg/L BAP, 0.05 mg/L GA_3, 0.02 mg/L NAA, 0.7% BD Difco™ agar; pH 5.7-5.8

12A3: MS, 3% sucrose, 1 mg/L thiamine HCl, 100 mg/L myo-inositol, 0.2 mg/L kinetin, 1 g/L AC, 0.7% BD Difco™ agar, pH 5.7-5.8

17N: 1/3 MS, 2% sucrose, 0.01 mg/L GA_3, 0.01 mg/L NAA, 0.7% BD Difco™ agar; pH 5.7-5.8
