# Supplementary material for: Adding Value to Cassava Genetic Resources Conserved at CIAT—Part I: A Review of Fifty Years of Collection, Conservation, Characterization and Distribution
Source: Plants (Basel). 2026 Jun 26;15(13):1981. doi: 10.3390/plants15131981 (PMC13363913; doi:10.3390/plants15131981)
Supplement: Supplementary file 1 [file plants-15-01981-s001.zip › Supplementary Table S5.pdf]

Supplementary Table S5. *Manihot* species held as herbarium specimens at CIAT.

Species	Samples	Location of collection
MANIHOT AESCULIFOLIA	1	OAXACA-MEXICO
MANIHOT ANGUSTILOBA	1	TOPOLOBAMPO-MEXICO
MANIHOT ANOMALA POHL	1	CASARES-BRASIL
MANIHOT ANOMALA POHL	1	TURVANIA-BRASIL
MANIHOT ANOMALA POHL	1	GUIABA-BRASIL
MANIHOT ANOMALA POHL	1	TAGUATINGA-BRASIL
MANIHOT ANOMALA POHL	1	ISRAELANDIA-BRASIL
MANIHOT CAERULESCENS	1	VILHENA-BRASIL
MANIHOT CAERULESCENS	1	TANQUINHOS-BRASIL
MANIHOT CAERULESCENS	1	COXIM-BRASIL
MANIHOT CAERULESCENS	1	MORRO-BRASIL
MANIHOT CAERULESCENS	1	DATAS-BRASIL
MANIHOT CAERULESCENS	1	AMARANTE-BRASIL
MANIHOT CAERULESCENS	1	CRISTOPOLIS-BRASIL
MANIHOT CARTHAGINENSIS	1	MORRO DO URUCUM-BRASIL
MANIHOT CARTHAGINENSIS	1	FERROVIARIA DO URUCUM-BRASIL
MANIHOT CARTHAGINENSIS	1	AURORA DO NORTE-BRASIL
MANIHOT CARTHAGINENSIS	1	BASE DE MORRO-BRASIL
MANIHOT CAUDATA	1	LAGO CHAPALA-MEXICO
MANIHOT CECROPIAEFOLIA	1	SERRANO-BRASIL
MANIHOT CRASSISEPALA	1	IZUCAR DE MATAMOROS-BRASIL
MANIHOT DAVISIAE	1	LOS MOCHIS -MEXICO
MANIHOT DIVERGENS	1	CERAMICA-BRASIL
MANIHOT EPRUINOSA	1	BELEM-BRASIL
MANIHOT EPRUINOSA	2	JUNCO DO SERIDO-BRASIL
MANIHOT EPRUINOSA	1	CERRO CORA-BRASIL
MANIHOT EPRUINOSA	1	BOM JESUS-BRASIL
MANIHOT EPRUINOSA	1	IRAUCUBA-BRASIL
MANIHOT EPRUINOSA	1	LAJES-BRASIL
MANIHOT EPRUINOSA	1	MONTE AZUL-BRASIL
MANIHOT CF EPRUINOSA	1	RIACHODE SANTANA -BRASIL
MANIHOT EPRUINOSA	1	PILOES-BRASIL
MANIHOT EPRUINOSA	2	IBOTIRAMA-BRASIL
MANIHOT EPRUINOSA	1	BARREIROS-BRASIL
MANIHOT EPRUINOSA	1	MARI-BRASIL
MANIHOT EPRUINOSA	1	POMEOS-BRASIL
MANIHOT EPRUINOSA	1	EUNAPOLIS-BRASIL
MANIHOT EPRUINOSA	1	JANAUBA-BRASIL
MANIHOT EPRUINOSA	2	GRAVATA-BRASIL
MANIHOT EPRUINOSA	1	CAMPO FORMOSO - BRASIL
MANIHOT EPRUINOSA	1	WENCESALAU GUIMARAES-BRASIL
MANIHOT ESCULENTA	1	AMELIA RODRIGUEZ-BRASIL

MANIHOT ESCULENTA	3	VILA BELA-BRASIL
MANIHOT ESCULENTA	1	COCOAL-BRASIL
MANIHOT ESCULENTA	1	GOIAS-BRASIL
MANIHOT ESCULENTA	2	NAZARIO-BRASIL
MANIHOT ESCULENTA	2	ANAPOLIS-BRASIL
MANIHOT ESCULENTA	1	ESTRELA DO NORTE-BRASIL
MANIHOT ESCULENTA	2	RONDONOPOLIS-BRASIL
MANIHOT ESCULENTA	2	PIMIENTA BUENO-BRASIL
MANIHOT ESCULENTA	3	PONTES E LACERDA-BRASIL
MANIHOT ESCULENTA	1	S.LUIS MONTES BELOS-BRASIL
MANIHOT ESCULENTA	1	ARIQUEMES-BRASIL
MANIHOT ESCULENTA	1	IPORA-BRASIL
MANIHOT ESCULENTA	1	PARAISO DO NORTE-BRASIL
MANIHOT ESCULENTA	1	CACOAL-BRASIL
MANIHOT ESCULENTA	1	PORTO VELHO-BRASIL
MANIHOT ESCULENTA	1	ARIQUEMES-BRASIL
MANIHOT ESCULENTA	1	VILLA RICA-BRASIL
MANIHOT ESCULENTA	1	ARIQUEMES-BRASIL
MANIHOT ESCULENTA	1	VILLA BELA-BRASIL
MANIHOT FLEMINGIANA	2	MIRANORTE-BRASIL
MANIHOT GABRIELENSIS	1	BRASIL
MANIHOT GLACILIS	2	PARQUE RURAL-BRASIL
MANIHOT GLACILIS	1	PONTE ALTA-BRASIL
MANIHOT GLACILIS	1	FORMOSA-BRASIL
MANIHOT GLACILIS	1	IPORA-BRASIL
MANIHOT GLAZIOVII	1	AMELIA RODRIGUEZ-BRASIL
MANIHOT GLAZIOVII	1	ITAPAJE-BRASIL
MANIHOT IRWINII	2	CORUMBA DE GOIAS-BRASIL
MANIHOT IRWINII	1	LUZIANIA-BRASIL
MANIHOT JACOBINENESIS	1	JACOBINA-BRASIL
MANIHOT MARACASENSIS	1	ANDARAI-BRASIL
MANIHOT MICHAELIS	1	CALIMA-MEXICO
MANIHOT MOSSAMEDENSIS TAUBERT	1	FORMOSA-BRASIL
MANIHOT MOSSAMEDENSIS TAUBERT	1	ISRAELANDIA-BRASIL
MANIHOT OAXACANA	1	RUINAS DE GUINGOLA-MEXICO
MANIHOT CF ORBICULARIS	1	ALIANCA-BRASIL
MANIHOT ORBICULARIS	2	NIQUELANDIA-BRASIL
MANIHOT PELTATA	1	CORUMBA DE GOIAS-BRASIL
MANIHOT PELTATA	1	ALTO PARAISO-BRASIL
MANIHOT PELTATA	1	CAVALGANTE-BRASIL
MANIHOT PELTATA	1	URUACU-BRASIL
MANIHOT PENTAPHYLLA	2	CORUMBA DE GOIAS-BRASIL
MANIHOT PENTAPHYLLA	1	CAVALGANTE-BRASIL
MANIHOT PENTAPHYLLA	1	MONTEALEGRE-BRASIL
MANIHOT PILOSA POHL	1	SANTA BARBARA-BRASIL

MANIHOT PILOSA POHL	1	BAREACENA-BRASIL
MANIHOT PILOSA POHL	1	SERRO-BRASIL
MANIHOT PRINGLEI	1	VICTORIA TAMANLIPAS-MEXICO
MANIHOT PRUINOSA	1	ISRAELANDIA-BRASIL
MANIHOT PRUINOSA	1	CAVALGANTE-BRASIL
MANIHOT PURPUREO	2	CAVALGANTE-BRASIL
MANIHOT PURPUREO	1	ALTO PARAISO-BRASIL
MANIHOT PUSILLA	1	GAMA-BRASIL
MANIHOT PSEUDOPRUINOSA	2	ALTO PARAISO-BRASIL
MANIHOT PSEUDOPRUINOSA	1	SAOJOAO DA ALIANCA-BRASIL
MANIHOT QUINQUELOBA	1	URUACU-BRASIL
MANIHOT QUINQUEPARTITA	3	CACOAL-BRASIL
MANIHOT QUINQUEPARTITA	1	PRESIDENTE MEDICI-BRASIL
MANIHOT QUINQUEPARTITA	3	PORTO VELHO-BRASIL
MANIHOT QUINQUEPARTITA	1	SANTA TEREZINHA-BRASIL
MANIHOT QUINQUEPARTITA	1	SANTANA DOARAGUAIA-BRASIL
MANIHOT RENIFORMIS POHL	1	MUCUGE-BRASIL
MANIHOT RABRICAULIS	1	DURANGO-MEXICO
MANIHOT SALICIFOLIA POHL	2	CAVALGANTE-BRASIL
MANIHOT SALICIFOLIA POHL	2	CORUMBA DE GOIAS-BRASIL
MANIHOT SAGITTATO	2	MONTEALEGRE-BRASIL
MANIHOT SPARSIFOLIA	1	CAVALGANTE-BRASIL
MANIHOT SPARSIFOLIA	1	TAGUATINGA-BRASIL
MANIHOT SPARSIFOLIA	2	BARTOLOMEU-BRASIL
MANIHOT SPARSIFOLIA	1	URUACU-BRASIL
MANIHOT SUBSPICATA	1	MONTERREY-MEXICO
MANIHOT TRIPARTITA	1	LAGOA SANTA-BRASIL
MANIHOT TRIPARTITA	1	IPORA-BRASIL
MANIHOT TRIPARTITA	1	BRASILIA-BRASIL
MANIHOT TRIPARTITA	1	FORMOSA-BRASIL
MANIHOT TRIPARTITA	1	CHAPADA DOS VEADEIROS-BRASIL
MANIHOT TRIPARTITA	1	CAMPOS BELOS-BRASIL
MANIHOT TRIPARTITA	2	PIRAPORA-BRASIL
MANIHOT TRIPARTITA	2	GOIAS-BRASIL
MANIHOT TRIPARTITA	1	URUACU-BRASIL
MANIHOT TRIPHYLLA POHL	1	ALEXANIA-BRASIL
MANIHOT TRIPHYLLA POHL	1	NORTE DE CRISTALINA-BRASIL
MANIHOT TRISTIS MULL	1	CUIABA-BRASIL
MANIHOT TRISTIS MULL	1	PIMENTA BUENO-BRASIL
MANIHOT TRISTIS MULL	1	CORUMBA DE GOIAS-BRASIL
MANIHOT TRISTIS MULL	2	GOIANIA-BRASIL
MANIHOT TRISTIS MULL	1	IPORA-BRASIL
MANIHOT TRISTIS MULL	1	GOIANAPOLIS-BRASIL
MANIHOT TRISTIS MULL	1	ITAUCU-BRASIL
MANIHOT TRISTIS MULL	2	URUACU-BRASIL

MANIHOT TRISTIS MULL	1	ANAPOLIS-BRASIL
MANIHOT TRISTIS MULL	1	CAVALCANTE-BRASIL
MANIHOT TRISTIS MULL	3	NIQUELANDIA-BRASIL
MANIHOT TRISTIS MULL	3	CAVALCANTE-BRASIL
MANIHOT TRISTIS MULL	1	ANAPOLIS-BRASIL
MANIHOT TRISTIS MULL	1	ARIQUEMES-BRASIL
MANIHOT TRISTIS MULL	1	OURO PRETO DO OESTE
MANIHOT TRISTIS MULL	1	PARAISO DO NORTE-BRASIL
MANIHOT TRISTIS MULL	1	VILLA RICA-BRASIL
MANIHOT TRISTIS MULL	1	CANARANA-BRASIL
MANIHOT TRISTIS MULL	1	ARIQUEMES-BRASIL
MANIHOT TRISTIS MULL	2	SAN ANTONIO DO DESCOBERTO-BRASIL
MANIHOT TRISTIS MULL	1	SAN ANTONIO DO DESCOBERTO-BRASIL
MANIHOT VIOLACEA POHL	1	CRISTALINA-BRASIL
MANIHOT VIOLACEA POHL	1	CORUMBA DE GOIAS-BRASIL
MANIHOT VIOLACEA POHL	1	GOIANIA-BRASIL
MANIHOT VIOLACEA POHL	1	GOIAS-BRASIL
MANIHOT VIOLACEA POHL	1	IPORA-BRASIL
MANIHOT VIOLACEA POHL	1	JARAGUA-BRASIL
MANIHOT VIOLACEA POHL	1	MUCUJE-BRASIL
MANIHOT WEBSTERAE	1	IZUCAR DE MATAMOROS-BRASIL

Total number of species: 46
Total number of samples: 188

Summary provided by Juan Jose Gonzales, CIAT, June 2026
