# Supplementary material for: Adding Value to Cassava Genetic Resources Conserved at CIAT—Part I: A Review of Fifty Years of Collection, Conservation, Characterization and Distribution
Source: Plants (Basel). 2026 Jun 26;15(13):1981. doi: 10.3390/plants15131981 (PMC13363913; doi:10.3390/plants15131981)
Supplement: Supplementary file 1 [file plants-15-01981-s001.zip › Supplementary Table S6.pdf]

Supplementary Table S6. Cassava morphological descriptors as defined by Fukuda et al. (2010) [6].

Descriptor names and timing of scoring*[a]	Basis for classification	General level of heritability[a]	Economic importance for growers or consumers[ab]
**Descriptors to be scored at three months after planting**			
*1. Color of apical leaves	Scale: light green to purple	High	Low
*2. Pubescence on apical leaves	Absent or present	High	Moderate
**Descriptors to be scored at six months after planting**			
3. Leaf retention	Scale: very poor to outstanding	Low	Moderate
*4. Shape of central leaflet	10 shape categories according to photos	High	Low
*5. Petiole color	Scale: light green to purple	High	Low
*6. Leaf color	Scale: light green to purple	High	Low
7. Number of leaf lobes	Number	High	Low
8. Length of leaf lobe	Central lobe, in cm	Medium	Low
9. Width of leaf lobe	Central lobe, widest point in cm	Medium	Low
*10. Ratio of lobe length to lobe	Central lobe, calculation	High	Low
width	Smooth vs winding (undulated)	High	Low
*11. Lobe margins	Middle third of plant, in cm	Medium	Low
12. Petiole length	Scale: green to red	High	Low
*13. Color of leaf vein	Scale: inclination upwards or downwards	Medium	Low
14. Orientation of the petiole	Absent or present	Medium	Low
15. Flowering	Absent or present	Medium	Low
16. Pollen			
**Descriptors to be scored at nine months after planting**			
17. Prominence of foliar scars	Scale: prominent or semi-prominent	High	Low
*18. Color of stem cortex	Categories: Orange; light or dark green	High	Low
*19. Color of stem epidermis	Categories: Cream, lt. or dk. brown,	High	Low
*20. Color of stem exterior	orange	High	Low
21. Distance between leaf scars	Categories: Lt. or dk. brown, silver, gray	Low	Low
*22. Growth habit of stem	Cm (middle third of plant)	High	Intermediate
23. Color of end branches of adult	Straight or zig-zag	High	Low
plant	Top 20 cm: scale - green to purple		
24. Length of stipules		Medium	Low
25. Stipule margin	Upper third of plant: long or short	High	Low
	Upper third of plant: entire or split		
**Descriptors to be scored at harvest**			
26. Fruit	Present or absent	Low	Low
27. Seed	Present or absent	Low	Low
28. Plant height	Cm	Medium	High
*29. Height to first branching	Cm	Med-High	High
*30. Levels of branching	Number	Medium	High
31. Branching habit	None; di-, tri-, or tetrachotomous	Medium	Medium
32. Angle of branching	At first branching level	Med-High	Medium
33. Shape of plant	Compact, open, umbrella, cylindrical	Medium	Medium

34. Number of storage roots/plant	Total storage roots per plant	Low	High
35. Number of commercial roots/plant	Commercial size roots per plant	Low	High
*36. Extent of root peduncle	Sessile, pedunculate, mixed	Med-High	Medium
37. Root constrictions	Scale: few to many	Low-Med	Medium
*38. Root shape	Category: conical, con-cyl, cylindrical,	Med-High	Medium
*39. External color of storage root	irreg.	High	Medium
*40. Color of root pulp (parenchyma)	White-cream, yellow, lt brown, dk brown	High	High
*41. Color of root cortex	White, cream, yellow, orange, pink[c]	High	Medium
*42. Cortex: ease of peeling	White or cream, yellow, pink, purple	Med-High	Medium
*43. Texture of root epidermis	Easy or difficult	Med-High	Low
44. Root taste (raw)	Smooth, intermediate, rough	Med-High	High
*45. Cortex thickness	Sweet, intermediate, bitter[d]	Med-High	Medium
46. Dry matter content	Thin, intermediate, thick	Med-High	High
47. Starch content	Estimate based on root density, or oven dry	Med-High	High
48. Harvest index	Estimate based on root density, or lab methods	Low-Med	Medium
49. Cyanogenic potential	Fresh root weight/(root weight + top weight)	Med-High	High
50. Postharvest deterioration	Scale 1-9 based on picrate method, or lab test	Very low	Medium
	Based on standard procedures (typically highly variable results)		

*Indicates priority for varietal identification and discrimination.

[a] Based on experience and suggestions of the author of the current report.

[b]Growers may associate these traits with preferred varieties, even if the trait is not actually genetically or physiologically related to specific traits of interest.

[c]Based on a more recent high level of interest in screening for beta-carotene levels, much more refined quantitative methodologies are available.

[d]In view of the potential for toxic effects of high levels of cyanogens, raw root tasting is not advised.
