# Supplementary material for: Adding Value to Cassava Genetic Resources Conserved at CIAT—Part I: A Review of Fifty Years of Collection, Conservation, Characterization and Distribution
Source: Plants (Basel). 2026 Jun 26;15(13):1981. doi: 10.3390/plants15131981 (PMC13363913; doi:10.3390/plants15131981)
Supplement: Supplementary file 1 [file plants-15-01981-s001.zip › Supplementary Table S7.pdf]

Supplementary Table S7. Putative simply inherited characters in cassava and their gene symbols.

Genetic condition	Cassava traits[a]	References
a/a	Albino seedlings (lethal)	Hershey and Ocampo, 1989 [78]
A/_	Normal chlorophyll	Hershey and Ocampo, 1989 [78]
z/z	Zigzag stems	Hershey and Ocampo, 1989 [78]
Z/_	Straight stems	Hershey and Ocampo, 1989 [78]
g/g	Dark green stem periderm	Hershey and Ocampo, 1989 [78]
G/_	Light green stem periderm	Hershey and Ocampo, 1989 [78]
y/y	White root parenchyma	Hershey and Ocampo, 1989 [78]
Y/y	Light yellow parenchyma	Hershey and Ocampo, 1989 [78]
Y/Y	Deep yellow parenchyma	Hershey and Ocampo, 1989 [78]
ms/ms	Male sterile	Jos and Nair, 1984 [80]
Ms/_	Male fertile	Jos and Nair, 1984 [80]
v/v	Broad leaf lobes	Graner, 1942 [79]
V/_	Narrow leaf lobes	Graner, 1942 [79]
m/m	White root epidermis	Graner, 1942 [79]
M/_	Dark root epidermis	Graner, 1942 [79]
p/p	Entire leaf margin	Hershey and Ocampo, 1989 [78]
P/_	Pandurate leaf margin	Hershey and Ocampo, 1989 [78]

[a]Probable epistatic effects of other genes.
