# Supplementary material for: Adding Value to Cassava Genetic Resources Conserved at CIAT—Part I: A Review of Fifty Years of Collection, Conservation, Characterization and Distribution
Source: Plants (Basel). 2026 Jun 26;15(13):1981. doi: 10.3390/plants15131981 (PMC13363913; doi:10.3390/plants15131981)
Supplement: Supplementary file 1 [file plants-15-01981-s001.zip › Supplementary Table S8.pdf]

Supplementary Table S8. Techniques and tools for molecular diversity analysis applied to the CIAT cassava collection.

Type of analysis	Targets	Materials and methods	What we learned about the collection	References
**TOOLS BASED ON BIOCHEMCIAL MARKERS:**				
**Isozymes**	12 isozyme systems	4304 accessions	Verify genetic integrity of in vitro accessions; duplicate identification	Ocampo et al., 1992 [81]
		Core collection	EST, PGM, PRX accounted for 47% of total variation; 57 alleles identified	Chavarriaga et al., 1999 [82]
**Metabolomics**	>400 differentiating chemical features[a]	481 genotypes (355 landraces)	Leaf, but not root, metabolome correlated with SNPs to define eco-geographical environments	Perez-Fons et al., 2023 [83]
**TOOLS BASED ON MOLECULAR MARKERS:**				
**RFLP**	Early diversity work	Wild relatives, landraces, African vs LAC materials	Early molecular basis for genetic diversity and *Manihot* phylogeny	Fregene et al., 1997 [84]
**RAPDs**	Genetic diversity and duplicate identification	Core collection	1.34% duplication in Core	Chavarriaga et al., 1999 [82]
**AFLP**	Measure similarity among accessions	35 accessions from core, 3 bred lines, 6 other taxa	Subsp. *flabellifolia* and *peruviana* are wild species most related to cassava	Roa et al., 1997 [4]
**SSR**	Duplicate identification	521 accessions	73 duplicates indicated	Chavarriaga et al. 1999 [82]
**SNP**	Simple, accurate and economical variety identification and diversity analysis	71,540 SNPs[a] 481 genotypes (355 landraces)	Combined with metabolomic data, clustering indicated 2 main and 7 sub-diversity centers in the Americas	Perez-Fons et al., 2023 [83]
		150 accessions; A 96-SNP panel (SNPY-CHIP)		Becerra et al., 2021 [85]
		66-SNP panel; About 10,000 samples, from Asia, Americas and Africa, from genebanks and farmers' fields	(Ongoing analysis); some very large clusters of duplicates due to sampling methods from farmers' fields (>1500)	Bohorquez, pers. comm

		5302 accessions	2518 unique genotypes; up to 84 duplicates per cluster	Carvajal et al. 2024 [10]
**SilicoDArT**	Methodology validation for cassava	Validated 38 accessions	Found high polymorphism suggesting advantages over previous tools	Xia et al., 2005 [86]
	Complete CIAT collection analysis	5302 accessions	2526 unique genotypes	Carvajal et al., 2024 [10]
**GBS**	Comparison of African and Latin American cassava genetic diversity	Subsamples from CIAT and IITA collections	Greater diversity in Latin American germplasm; not a significant bottleneck in introduction to Africa	Ferguson et al., 2012, 2019 [87, 88]

[a]Metabolomic and SNP studies combined for diversity analysis.
